# *Be*fragung zu den Arbeits-, *W*eiterbildungs- und Forsch*u*ngsbedingungen von A*ss*istenzärztinnen und -ärzten in der internistisch-rheumatologischen Wei*t*erbildung – BEWUSST

**DOI:** 10.1007/s00393-023-01395-6

**Published:** 2023-08-11

**Authors:** Fabian Proft, die Kommission Fort- und Weiterbildung der Deutschen Gesellschaft für Rheumatologie, Diana Vossen, Xenofon Baraliakos, Michael N. Berliner, Martin Fleck, Gernot Keyßer, Andreas Krause, Hanns-Martin Lorenz, Bernhard Manger, Florian Schuch, Christof Specker, Jürgen Wollenhaupt, Anna Voormann, Matthias Raspe, Martin Krusche, Alexander Pfeil

**Affiliations:** 1 Abteilung für Rheumatologie, Medizinische Klinik für Gastroenterologie, Infektiologie und Rheumatologie, Campus Benjamin Franklin, Charité Universitätsmedizin, Berlin, Deutschland; 2 Rheumazentrum Ruhrgebiet, Ruhr-Universität Bochum, Herne, Deutschland; 3 Rheumatologie und Geriatrie, Helios Klinikum Berlin-Buch, Berlin, Deutschland; 4 Klinik und Poliklinik für Innere Medizin I, Universitätsklinikum Regensburg, Regenburg, Deutschland; 5 Klinik und Poliklinik für Rheumatologie/Klinische Immunologie, Asklepios Klinikum Bad Abbach, Bad Abbach, Deutschland; 6 Department für Innere Medizin, Klinik für Innere Medizin II, Universitätsklinikum Halle, Halle (Saale), Deutschland; 7 Klinik für Innere Medizin, Abteilung Rheumatologie, klinische Immunologie und Osteologie, Immanuel Krankenhaus Berlin, Berlin, Deutschland; 8 Sektion Rheumatologie, Medizinische Klinik V, Universitätsklinikum Heidelberg, Heidelberg, Deutschland; 9 Medizinische Klinik 3, Rheumatologie und Immunologie, Universitätsklinikum Erlangen, Friedrich-Alexander Universität Erlangen-Nürnberg, Erlangen, Deutschland; 10 Internistische Praxisgemeinschaft Rheumatologie – Nephrologie, Erlangen, Deutschland; 11 Klinik für Rheumatologie und Klinische Immunologie, Evangelisches Krankenhaus Kliniken Essen-Mitte, Essen, Deutschland; 12 Immunologikum Hamburg, Hamburg, Deutschland; 13 Deutsche Gesellschaft für Rheumatologie, Berlin, Deutschland; 14 Klinik für Pneumologie, Beatmungsmedizin und Intensivmedizin mit dem Arbeitsbereich Schlafmedizin Charité – Campus Virchow-Klinikum, Charité – Universitätsmedizin, Berlin, Deutschland; 15 Sektion für Rheumatologie und Entzündliche Systemerkrankungen, Universitätsklinikum Hamburg-Eppendorf (UKE), Hamburg, Deutschland; 16 Klinik für Innere Medizin III, Universitätsklinikum Jena, Friedrich-Schiller-Universität Jena, Am Klinikum 1, 07747 Jena, Deutschland

**Keywords:** Fachgebiet Innere Medizin und Rheumatologie, Vereinbarkeit von Beruf und Familie, Vereinbarkeit von Arbeit und Forschung, Perspektive, Tätigkeitsfelder, Speciality of internal medicine and rheumatology, Compatibility of career and family, Compatibility of work and research, Perspectives, Fields of activity

## Abstract

**Hintergrund:**

Daten zur Aus- und Weiterbildungssituation von Assistenzärzt:innen des Fachgebietes Innere Medizin und Rheumatologie liegen für das Bundesgebiet nicht vor. Aus diesem Grund initiierte die Kommission Fort- und Weiterbildung der Deutschen Gesellschaft für Rheumatologie (DGRh) eine Umfrage zu den Arbeits‑, Weiterbildungs- und Forschungsbedingungen von Assistenzärztinnen und -ärzten in der rheumatologischen Weiterbildung.

**Methodik:**

Es wurden 102 Fragen zu den Themenkomplexen Arbeitsbedingungen im Berufsalltag, ärztliche Fort- und Weiterbildung, Vereinbarkeit von Beruf und Familie, Vereinbarkeit von Arbeit und Forschung, Perspektive als Rheumatolog:in und praktische Tätigkeiten über einen Online-Fragebogen gestellt.

**Ergebnisse:**

Insgesamt haben sich 102 Teilnehmer:innen an der Umfrage beteiligt; 48,1 % der Befragten waren mit der beruflichen Situation zufrieden, 40,2 % der Teilnehmer:innen wurden durch eine/n fachärztliche/n Mentor:in betreut, und 54,9 % der Teilnehmer:innen sind wissenschaftlich tätig. Eine Vereinbarkeit von Familie und Beruf war für 34,7 % möglich. Nach Abschluss der Facharztausbildung strebten 52,9 % der Befragten eine kombinierte klinische und ambulante Tätigkeit an.

**Diskussion:**

Die Hälfte der angehenden Rheumatolog:innen ist mit der beruflichen Tätigkeit zufrieden, wobei das Mentoring der Weiterbildungsassistent:innen eine weitere Verbesserung erfahren sollte. Hinsichtlich der gewünschten kombinierten klinischen und ambulanten Tätigkeit sollten die vorhandenen Optionen ausgebaut bzw. neue berufliche Tätigkeitsfelder etabliert werden, damit das Fachgebiet für den Nachwuchs attraktiv bleibt.

**Zusatzmaterial online:**

Die Online-Version dieses Beitrags (10.1007/s00393-023-01395-6) enthält weiterführende Informationen zur Befragung zu den Arbeits‑, Weiterbildungs- und Forschungsbedingungen von Assistenzärztinnen und -ärzten in der internistisch-rheumatologischen Weiterbildung – BEWUSST.

## Abstract (en)

**Background:**

Data on the training and continuing education situation of residents in the field of internal medicine and rheumatology are not available for Germany. For this reason, the Commission for Education and Training of the German Society of Rheumatology (DGRh) initiated the BEWUSST survey on the working, training and research conditions of residents in rheumatology.

**Methods:**

A total of 102 questions on the topics of working conditions in everyday professional life, continuing medical education and training, compatibility of career and family, compatibility of work and research, perspectives as a rheumatologist and practical activities were included in an online questionnaire.

**Results:**

A total of 102 participants took part in the survey. Of the respondents 48.1% were satisfied with their professional situation, 40.2% of the participants were supervised by a specialist mentor and 54.9% were working as scientists during their work as a physician. A compatibility of family and career was possible for 34.7%. After completion of the residency 52.9% of the respondents aspired to a combined clinical and outpatient activity.

**Conclusion:**

Half of the trainee rheumatologists are satisfied with their professional activities, although mentoring of the assistants in training should be further improved. With respect to the desired combined clinical and outpatient activity, the existing options should be expanded or new professional fields of activity should be established, so that the specialty remains attractive for the upcoming generations.

In den vergangenen Jahren hat das Fachgebiet der Rheumatologie und klinischen Immunologie enorme Fortschritte in Forschung, Klinik und Therapie erlebt. Durch ein besseres Verständnis der pathophysiologischen Vorgänge im Immunsystem konnte eine Vielzahl von neuen innovativen Therapien entwickelt werden. Weiterhin können durch eine zeitnahe Diagnostik und entsprechende Therapieeinleitung potenziell immer bessere Therapieerfolge erzielt und bleibende Schäden für die Patient:innen vermieden werden.

Jedoch steht die deutsche Rheumatologie in den kommenden Jahren, ähnlich wie andere medizinische Teilgebiete, vor besonderen Herausforderungen: Aufgrund des sich absehbar erhöhenden Versorgungsbedarfes bei sich weiterhin verschärfendem Fachkräftemangel drohen hier zunehmende Versorgungsengpässe. Bereits im Memorandum der Deutschen Gesellschaft für Rheumatologie (DGRh) von 2016 wurde eine deutliche Steigerung der Stellen für rheumatologische Fachärzt:innen zur Bedarfsdeckung gefordert [18].

Seitdem hat sich jedoch strukturell leider wenig geändert, und die Zahl an neuen Fachärzt:innenstellen stagniert [14]. Eine Umfrage in Sachsen-Anhalt, Sachsen und Thüringen von Keyßer et al. ergab, dass in den nächsten 10 Jahren die Hälfte der Rheumatolog:innen das Pensionsalter erreicht [4]. Im Hinblick auf die steigende Prävalenz der Erkrankungen und die höhere Lebenserwartung der Patienten [6] wird der Bedarf an Rheumatolog:innen in Deutschland somit noch weiter zunehmen [1].

In einer Analyse im Jahr 2021 konnte gezeigt werden, dass insgesamt 17,2 % der Aus- und Weiterbildungsstellen im Fachgebiet unbesetzt blieben, was zum größten Teil den ambulanten Sektor (43,1 %, klinischer Bereich 11,4 %) mit einer fehlenden Finanzierung betrifft [12]. Diese Lücke gilt es zu schließen, um diesem Abwärtstrend entgegenzuwirken.

Hierzu bedarf es unter anderem attraktiver Aus- und Weiterbildungsbedingungen, die sich an einheitlichen Standards orientieren [13] und Möglichkeiten für moderne Arbeitszeit- und Karrieremodelle bieten. Erste kleinere Arbeiten aus den vergangenen Jahren konnten hier bereits Verbesserungspotenzial aufzeigen [5, 10].

Um die aktuelle Aus- und Weiterbildungssituation systematisch zu analysieren und potenzielle Verbesserungsmöglichkeiten zu identifizieren, initiierte die Kommission Fort- und Weiterbildung der DGRh daher im Jahr 2022 die BEWUSST-Umfrage (*Be*fragung zu den Arbeits-, *W*eiterbildungs- und Forsch*u*ngsbedingungen von A*ss*istenzärztinnen und -ärzten in der internistisch-rheumatologischen Wei*t*erbildung – BEWUSST). Für eine bessere Vergleichbarkeit mit anderen Fachdisziplinen wurde als Grundlage die Umfragen von Raspe et al. der Deutschen Gesellschaft für Innere Medizin (DGIM) herangezogen [15, 16]. Hier wurde im Jahr 2016 eine Erfassung der Weiterbildbildungssituation in der allgemeinen Inneren Medizin durchgeführt.

Darüber hinaus adressiert die BEWUSST-Umfrage auch noch weitere fachspezifische Aspekte des rheumatologischen Weiterbildungscurriculums, wie z. B. die immunologische Labordiagnostik und rheumatologische Untersuchungstechniken.

## Methoden

Der Fragebogen wurde mit dem System QuestionPro (QuestionPro GmbH, Berlin, Deutschland) erstellt. Alle Fragen konnten im Zeitraum vom 04.05.2022 bis 31.10.2022 online beantwortet werden. Die Umfrage wurde über den Newsletter der DGRh sowie über die sozialen Netzwerke der Arbeitsgemeinschaft Junge Rheumatologie (AGJR) beworben. Alle weiterbildungsbefugten Rheumatolog:innen erhielten ein separates Anschreiben, mit dem auf die Umfrage hingewiesen und die Bitte geäußert wurde, die Weiterzubildenden auf die Umfrage aufmerksam zu machen und für die Teilnahme zu motivieren.

Die Umfrage umfasste insgesamt 102 Fragen zu folgenden Themenbereichen: Basisdaten (*n* = 15), Arbeitsbedingungen im Berufsalltag (*n* = 5), ärztliche Fort- und Weiterbildung (*n* = 17), Vereinbarkeit von Beruf und Familie (*n* = 10), Vereinbarkeit von Arbeit und Forschung (*n* = 16), Perspektive als Rheumatologin/Rheumatologe (*n* = 14), praktische Tätigkeiten (*n* = 22) und persönliche Meinungen/Kommentare (*n* = 3).

Nach Abschluss der Evaluation wurden die Umfrageergebnisse aus dem System QuestionPro extrahiert und in eine Excel-Tabelle (Microsoft Excel 2016, Redmond, WA, USA) überführt. Alle Angaben lagen als absolute Anzahl bzw. Prozentwert vor.

### Ethik

Die retrospektive Datenauswertung erfolgte entsprechend den Regularien des Ethikkomitees am Universitätsklinikum Jena.

### Statistik

Nach Zusammenfassung der Daten erfolgte die Durchführung einer deskriptiven Statistik. Die statistischen Analysen wurden mit der Software IBM SPSS Statistics Version 27.0 (IBM SPSS Statistics, Chicago, IL, USA) für Windows durchgeführt.

## Ergebnisse

### Basisdaten

An der Umfrage haben insgesamt 102 Weiterzubildende (Frauen: *n* = 68 [66,7 %] und Männer *n* = 34 [33,3 %]) teilgenommen. Die Hauptaltersspanne betrug 30 bis 34 Jahre (40,2 %). Der Hauptteil der Teilnehmer:innen war in den Bundesländern Bayern (20,6 %) und Nordrhein-Westfalen (19,6 %) tätig. Bei 38,2 % der Befragten lebten 2 Kinder im Haushalt; 91,2 % der Teilnehmer:innen wiesen keine Facharztbezeichnung auf.

**Figure 1:**
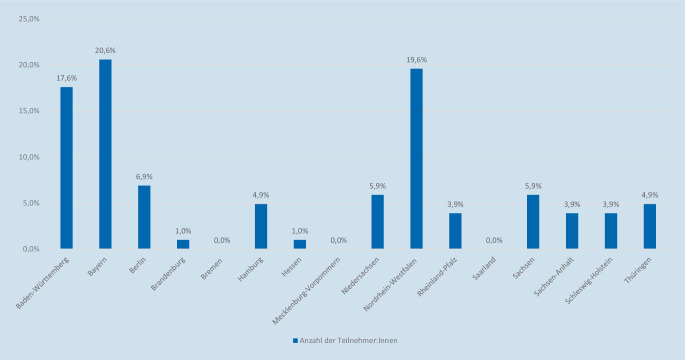

Insgesamt 46,1 % bzw. 33,3 % der Teilnehmer:innen waren auf einer Normalstation bzw. in einer Klinikambulanz tätig. Bei 71,6 % der Befragten befand sich der Klinikträger in einer öffentlichen Trägerschaft; 47,1 % arbeiteten an einem Universitätsklinikum. Als primäres Karriereziel strebten 38,1 % der Teilnehmer:innen eine Niederlassung, 27,3 % eine Oberärzt:innenfunktion an einem Krankenhaus, 16,5 % eine akademische Laufbahn mit Habilitation/Professur und 3,4 % eine Chefärzt:innenfunktion an (Abb. 1 und Tab. 1).

**Table 1:** 

Teilnehmer	Gesamt	*n* = 102
	Frauen	*n* = 68 (66,7 %)
	Männer	*n* = 34 (33,3 %)
	Divers	*n* = 0 (0 %)
Alter	20 bis 24 Jahre	*n* = 0 (0 %)
	25 bis 29 Jahre	*n* = 23 (22,6 %)
	30 bis 34 Jahre	*n* = 41 (40,2 %)
	35 bis 39 Jahre	*n* = 31 (30,4 %)
	40 bis 44 Jahre	*n* = 3 (2,9 %)
	> 45 Jahre	*n* = 4 (3,9 %)

### Arbeitsbedingungen im Berufsalltag

Mit der beruflichen Situation waren 36,3 % bzw. 11,8 % der Teilnehmer:innen „eher zufrieden“ bzw. „sehr zufrieden“. Als Faktoren, die mit einer Unzufriedenheit verbunden waren, wurde am häufigsten eine hohe zeitliche Arbeitsbelastung (14,6 %) ohne exakte Zeitdefinition genannt. Der Anteil der Tätigkeiten am beruflichen Alltag gliedert sich wie folgt: 42,7 % Arbeiten mit und am Patienten, 31,8 % patientenbezogene Arbeiten und 25,5 % nichtärztliche bzw. patientenferne Tätigkeiten (Tab. 2).

**Table 2:** 

Fragen	Mögliche Antwort	Anzahl (Prozent)
Wie zufrieden sind Sie insgesamt mit Ihrer augenblicklichen beruflichen Situation?	Sehr unzufrieden	*n* = 10 (9,8 %)
	Eher unzufrieden	*n* = 17 (16,7 %)
	Unentschieden – teils, teils	*n* = 26 (25,4 %)
	Eher zufrieden	*n* = 37 (36,3 %)
	Sehr zufrieden	*n* = 12 (11,8 %)
Welche Faktoren sorgen bei Ihnen für Unzufriedenheit? (bis zu 3 Antworten möglich)	Hohe zeitliche Arbeitsbelastung	*n* = 41 (13,8 %)
	Unregelmäßige Arbeitszeiten und Schichtdient	*n* = 13 (4,4 %)
	Hohe Arbeitsverdichtung	*n* = 26 (8,8 %)
	Schlechte Vereinbarkeit von Familie und Beruf	*n* = 13 (4,4 %)
	Keine Zeit für Forschung	*n* = 17 (5,7 %)
	Mein Privatleben leidet	*n* = 21 (7,1 %)
	Keine eigenständigen medizinischen Entscheidungen möglich	*n* = 2 (0,7 %)
	Starker ökonomischer Einfluss auf medizinische Entscheidungen	*n* = 16 (5,4 %)
	Schlechtes Arbeitsklima	*n* = 5 (1,7%)
	Starre Hierarchien	*n* = 4 (1,3 %)
	Hoher Anteil arztfremder Tätigkeiten	*n* = 35 (11,8 %)
	Mangelnde Qualität der medizinischen Weiterbildung	*n* = 13 (4,4 %)
	Fehlende Anleitung und Supervision	*n* = 19 (6,4 %)
	Geringe Anerkennung für die geleistete Arbeit	*n* = 13 (4,4 %)
	Schlechte Organisation der Abläufe/schlechte Absprachen	*n* = 26 (8,8 %)
	Mangelnde Zukunftsperspektive	*n* = 26 (8,8 %)
	Sonstiges: Freitext	*n* = 7 (2,4 %)

### Ärztliche Fort- und Weiterbildung

Insgesamt 75,5 % der Teilnehmer:innen arbeiteten in Vollzeit und 24,5 % in Teilzeit. Benachteiligt im Fortkommen ihrer Weiterbildung fühlten sich 60 % der in Teilzeit tätigen Kolleg:innen. An einer Einrichtung mit voller Weiterbildungsermächtigung für den Facharzt bzw. die Fachärztin für Innere Medizin und Rheumatologie waren 81,4 % der Teilnehmer:innen tätig; 36,3 % der Befragten hatten primär einen Arbeitsvertrag über die gesamte Weiterbildungszeit erhalten; 22,5 % gaben an, dass an ihrer Weiterbildungsstätte ein strukturiertes Weiterbildungscurriculum vorliegt und eine transparente Darstellung der Rotationen vonseiten der Klinikleitung (42,2 %) erfolgt. Die Weiterbildung schlossen 68,6 % der Teilnehmer:innen in der vorgesehenen Weiterbildungszeit ab. Die Inanspruchnahme externer Weiterbildungsangebote (55,9 %) wurde als hilfreich angesehen (Tab. 3).

**Table 3:** 

Fragen	Mögliche Antwort	Anzahl (Prozent)
Arbeiten Sie derzeit in Vollzeit oder in Teilzeit?	Vollzeit	*n* = 77 (75,5 %)
	Teilzeit	*n* = 25 (24,5 %)
Gibt es bei Ihnen ein strukturiertes Weiterbildungscurriculum mit von Beginn an fest geplanten Lerninhalten/Rotationen?	Nein	*n* = 79 (77,5 %)
	Ja	*n* = 23 (22,5 %)
Werden Sie Ihre Weiterbildung in der vorgesehen Regelweiterbildungszeit abschließen können (z. B. 5 Jahre für den FA Innere Medizin)?	Nein	*n* = 32 (31,4 %)
	Ja	*n* = 70 (68,6 %)

*FA* Facharzt

### Vereinbarkeit von Beruf und Familie

Bezüglich der Zufriedenheit von der Vereinbarkeit von Familie und Beruf wurden folgende Ergebnisse erhoben: 6,9 % „trifft voll zu“, 27,8 % „trifft eher zu“, 29,2 % „trifft teils zu“ und 22,2 % „trifft eher nicht zu“. Bezüglich der Verschiebung der Aufgaben zugunsten der Arbeit (23,6 % „trifft voll zu“, 40,3 % „trifft eher zu“ und 23,6 % „trifft teils zu“) und der Unterstützung durch die Kolleg:innen (12,5 % „trifft voll zu“, 36,1 % „trifft eher zu“ und 29,2 % „trifft teils zu“) ergibt sich ein ähnliches Bild. Die flexible Gestaltung von Arbeitszeiten (21,4 %), Homeoffice (19,9 %), weniger Überstunden (19,4 %), die bessere Planung von Arbeitszeiten (14,4 %) und ein Kinderbetreuungsplatz (4,5 %) bzw. eine Ganztagsbetreuung (4,0 %) führten laut der Teilnehmer:innen zur Verbesserung der Balance zwischen Arbeit und dem Privatleben (Tab. 4).

**Table 4:** 

Fragen	Mögliche Antwort	Anzahl (Prozent)
Die folgenden Fragen zu Vereinbarkeit von Beruf und Familie richten sich in erster Linie an Ärztinnen und Ärzte mit Kind/Kindern. Aber auch wenn Sie kein Kind/keine Kinder haben, können Sie die nachfolgenden Fragen sehr gerne beantworten	Ich habe ein Kind/Kinder	*n* = 37 (36,3 %)
	Ich habe kein Kind/Kinder, möchte die Fragen zu Beruf und Familie dennoch beantworten	*n* = 35 (34,3 %)
	Ich habe kein Kind/Kinder und möchte die Fragen zu Familie und Beruf überspringen	*n* = 30 (29,4 %)
Ich bin zufrieden mit der Vereinbarkeit von Familie und Beruf an meinem Arbeitsplatz	Trifft voll zu	*n* = 5 (6,9 %)
	Trifft eher zu	*n* = 20 (27,8 %)
	Teils, teils	*n* = 21 (29,2 %)
	Trifft eher nicht zu	*n* = 16 (22,2 %)
	Trifft nicht zu	*n* = 10 (13,9 %)
Rücksicht auf Mitarbeiter mit familiären Verpflichtungen geht häufig zulasten von Mitarbeitern ohne familiäre Verpflichtungen	Trifft voll zu	*n* = 29 (40,3 %)
	Trifft eher zu	*n* = 20 (27,8 %)
	Trifft teils zu	*n* = 16 (22,2 %)
	Trifft eher nicht zu	*n* = 4 (5,6 %)
	Trifft nicht zu	*n* = 3 (4,2 %)
Haben Sie Elternzeit genommen?	Ich habe bisher keine Elternzeit genommen	*n* = 41 (56,9 %)
	Ich habe bereits Elternzeit genommen	*n* = 31 (43,1 %)
Welche der folgenden Faktoren würden Ihnen eine gute Balance zwischen Arbeit und einem Privatleben mit familiären Aufgaben erleichtern/ermöglichen? (Auswahl der 3 wichtigsten Faktoren)	Flexiblere Gestaltung der Arbeitszeiten z. B. durch mehr Mitsprache bei der Festlegung der Arbeitszeit	*n* = 43 (21,7 %)
	Besser planbare oder regelmäßigere Arbeitszeiten	*n* = 29 (14,6 %)
	Weniger Überstunden	*n* = 39 (19,7 %)
	Kinderbetreuungsplatz	*n* = 9 (4,5 %)
	Ganztagsbetreuung	*n* = 8 (4,0 %)
	Kinderbetreuungsplatz in der Nähe der Arbeitsstelle	*n* = 4 (2,0 %)
	Mehr Rücksicht von Kolleginnen und Kollegen	*n* = 2 (1,0 %)
	Teile der Arbeit zu Hause erledigen (Dokumentation, Arztbriefe über Intranet/VPN)	*n* = 40 (20,2 %)
	Optionaler Betreuungsdienst für Kinder bzw. Angehörige in Notfällen, bei Sitzungen oder während der Schulferien	*n* = 9 (4,5 %)
	Finanzielle Unterstützung	*n* = 4 (2,0 %)
	Mentorenprogramm	*n* = 5 (2,5 %)
	Ich bin mit dem derzeitigen Angebot zufrieden	*n* = 6 (3,0 %)

*VPN* Virtual Private Network

### Vereinbarkeit von Arbeit und Forschung

Insgesamt waren 69,6 % der Teilnehmer:innen promoviert, wobei von diesen 54,9 % weiterhin wissenschaftlich tätig waren. Die wissenschaftlichen Arbeiten beschäftigten sich v. a. mit klinisch orientierten Themen (73 %). Primär wurde die wissenschaftliche Arbeit in der Freizeit (71,6 %) durchgeführt. Nur 26,4 % waren in der Arbeitszeit wissenschaftlich tätig. Im Durchschnitt sind die Teilnehmer:innen auf einem wissenschaftlichen Beitrag je Kongress als Koautor aufgeführt worden. Eine Habilitation wurde von 28,4 % der Befragten angestrebt. Des Weiteren waren 61,8 % der Teilnehmer:innen in die studentische Lehrtätigkeit eingebunden (Tab. 5).

**Table 5:** 

Fragen	Mögliche Antwort	Anzahl (Prozent)
Sind Sie derzeit wissenschaftlich tätig oder wollen dies in Zukunft sein?	Nein	*n* = 46 (45,1 %)
	Ja	*n* = 56 (54,9 %)
Mit welchem wissenschaftlichen Gebiet befasst sich Ihre Forschungsarbeit? (Mehrfachnennung möglich)	Experimentell (Grundlagenforschung)	*n* = 21 (18,3 %)
	Klinisch orientiert	*n* = 84 (73,0 %)
	Epidemiologisch	*n* = 10 (8,7 %)
Wann führen Sie Ihre wissenschaftliche Arbeit durch?	Während der Arbeitszeit, neben der klinischen Tätigkeit	*n* = 27 (26,4 %)
	In der Freizeit im Anschluss an die klinische Tätigkeit	*n* = 73 (71,6 %)
	Es besteht eine Freistellung zur Durchführung der Forschungsarbeit	*n* = 2 (2,0 %)
Sind Sie an der studentischen Lehre beteiligt?	Nein	*n* = 39 (38,2 %)
	Ja	*n* = 63 (61,8 %)

### Perspektive Rheumatologe/Rheumatologin

Für 77,5 % war eine Ausbildung im ambulanten Bereich von Interesse. Im niedergelassenen Bereich absolvierten 13,7 % der Befragten einen Ausbildungsabschnitt. Nach Abschluss der Facharztausbildung strebten 52,9 % eine kombinierte klinische und ambulante Tätigkeit an; 64 % hatten vor, im Angestelltenverhältnis tätig zu sein. Für 74,5 % stellte die Tätigkeit in einem medizinischen Versorgungszentrum als angestellte Ärztin oder Arzt eine Option dar; 82,4 % der befragten Kolleg:innen hatten noch keinen Kontakt zu einer niedergelassenen Rheumatologin oder einem niedergelassenen Rheumatologen (Tab. 6 und 7).

**Table 6:** 

Fragen	Mögliche Antwort	Anzahl (Prozent)
Ist eine Facharztausbildung im ambulanten Bereich von Interesse?	Nein	*n* = 23 (22,5 %)
	Ja	*n* = 79 (77,5 %)
Wurden bereits Ausbildungsabschnitte im niedergelassenen Bereich absolviert?	Nein	*n* = 88 (86,3 %)
	Ja	*n* = 14 (13,7 %)
Streben Sie eine klinische oder ambulante Tätigkeit an?	Klinik	*n* = 13 (12,7 %)
	Ambulante Tätigkeit	*n* = 35 (34,3 %)
	Klinik und ambulante Tätigkeit	*n* = 54 (52,9 %)

**Table 7:** 

Fragen	Mögliche Antwort	Anzahl (Prozent)
**Work-Life-Balance**	1 (sehr wichtig)	*n* = 38 (37,3 %)
	2	*n* = 33 (32,4 %)
	3	*n* = 9 (8,8 %)
	4	*n* = 8 (7,8 %)
	5	*n* = 5 (4,9 %)
	6	*n* = 5 (4,9 %)
	7	*n* = 4 (3,9 %)
	8 (unwichtig)	*n* = 0 (0 %)
**Teilzeittätigkeit**	1 (sehr wichtig)	*n* = 16 (15,7 %)
	2	*n* = 12 (11,8 %)
	3	*n* = 11 (10,8 %)
	4	*n* = 12 (11,8 %)
	5	*n* = 13 (12,7 %)
	6	*n* = 12 (11,8 %)
	7	*n* = 9 (8,8 %)
	8 (unwichtig)	*n* = 17 (16,7 %)
**Vereinbarkeit von Familie und Beruf**	1 (sehr wichtig)	*n* = 44 (43,1 %)
	2	*n* = 28 (27,5 %)
	3	*n* = 9 (8,8 %)
	4	*n* = 8 (7,8 %)
	5	*n* = 5 (4,9 %)
	6	*n* = 3 (2,9 %)
	7	*n* = 5 (4,9 %)
	8 (unwichtig)	*n* = 0 (0 %)
**Klinische Tätigkeit**	1 (sehr wichtig)	*n* = 26 (25,5 %)
	2	*n* = 20 (19,6 %)
	3	*n* = 22 (21,6 %)
	4	*n* = 12 (11,8 %)
	5	*n* = 5 (4,9 %)
	6	*n* = 8 (7,8 %)
	7	*n* = 8 (7,8 %)
	8 (unwichtig)	*n* = 1 (1,0 %)
**Selbstständigkeit**	1 (sehr wichtig)	*n* = 14 (13,7 %)
	2	*n* = 20 (19,6 %)
	3	*n* = 18 (17,6 %)
	4	*n* = 13 (12,7 %)
	5	*n* = 10 (9,8 %)
	6	*n* = 11 (10,8 %)
	7	*n* = 7 (6,9 %)
	8 (unwichtig)	*n* = 9 (8,8 %)
**Regionale Bindung**	1 (sehr wichtig)	*n* = 14 (13,7 %)
	2	*n* = 13 (12,7 %)
	3	*n* = 16 (15,7 %)
	4	*n* = 17 (16,7 %)
	5	*n* = 12 (11,8 %)
	6	*n* = 11 (10,8 %)
	7	*n* = 10 (9,8 %)
	8 (unwichtig)	*n* = 9 (8,8 %)
**Keinen Wochenend‑/Nachtdienst**	1 (sehr wichtig)	*n* = 39 (38,2 %)
	2	*n* = 22 (21,6 %)
	3	*n* = 11 (10,8 %)
	4	*n* = 9 (8,8 %)
	5	*n* = 5 (4,9 %)
	6	*n* = 6 (5,9 %)
	7	*n* = 1 (1,0 %)
	8 (unwichtig)	*n* = 9 (8,8 %)
**Verdienstmöglichkeiten**	1 (sehr wichtig)	*n* = 23 (22,5 %)
	2	*n* = 26 (25,5 %)
	3	*n* = 16 (15,7 %)
	4	*n* = 16 (15,7 %)
	5	*n* = 12 (11,8 %)
	6	*n* = 1 (1,0 %)
	7	*n* = 4 (3,9 %)
	8 (unwichtig)	*n* = 4 (3,9 %)

### Praktische Tätigkeiten

Zum Zeitpunkt der Befragung hatten 91,2 % der Teilnehmer:innen die Facharztweiterbildung noch nicht abgeschlossen; 40,2 % der Teilnehmer:innen wurden durch eine/n Mentor:in (Fachärztin bzw. Facharzt für Innere Medizin und Rheumatologie) am Arbeitsplatz betreut. Unter Anleitung bzw. selbstständig führten 41,2 % bzw. 86,3 % der Kolleg:innen Arthrosonographien durch. Für die Gefäßsonographien wurden folgende Daten erhoben: Gefäßsonographien unter Anleitung 35,3 % und selbstständige Gefäßsonographien 43,1 %; 60,8 % bzw. 73,5 % der Teilnehmer:innen konnten Gelenkpunktion unter Anleitung bzw. selbstständig anwenden. Hinsichtlich der Kapillarmikroskopie führten 29,4 % der Kolleg:innen die Untersuchung unter einer Anleitung durch (54,9 % selbstständig). Bei 53,9 % der Befragten bestand die Möglichkeit zum Erwerb der Handlungskompetenz für die rheumatologische/immunologische Labordiagnostik an der eigenen Weiterbildungsstätte, und 30,4 % der Teilnehmer:innen besaßen nach eigener Einschätzung zum Zeitpunkt der Befragung bereits die Handlungskompetenz für die Labordiagnostik. Bezüglich der bildgebenden radiologischen bzw. nuklearmedizinischen Verfahren hatten die Befragten für folgende Verfahren bereits eine Ausbildung erhalten: 48,0 % Röntgendiagnostik, 35,3 % Computertomographie, 38,2 % Magnetresonanztomographie und 29,4 % nuklearmedizinisch-bildgebende Verfahren (Tab. 8).

**Table 8:** 

Fragen	Mögliche Antwort	Anzahl (Prozent)
Werden praktische Tätigkeiten am Arbeitsplatz durch einen Mentor (Facharzt) betreut?	Nein	*n* = 41 (40,2 %)
	Ja	*n* = 61 (59,8 %)
Führen Sie selbstständig Arthrosonographien durch?	Nein	*n* = 14 (13,7 %)
	Ja	*n* = 88 (86,3 %)
Führen Sie selbstständig Gefäßsonographien durch?	Nein	*n* = 58 (56,9 %)
	Ja	*n* = 44 (43,1 %)
Führen Sie selbstständig Gelenkpunktionen durch?	Nein	*n* = 27 (26,5 %)
	Ja	*n* = 75 (73,5 %)
Führen Sie selbstständig Kapillarmikroskopien durch?	Nein	*n* = 46 (45,1 %)
	Ja	*n* = 56 (54,9 %)

## Diskussion

Die BEWUSST-Umfrage befasst sich mit den Arbeits‑, Weiterbildungs- und Forschungsbedingungen von Assistenzärztinnen und -ärzten in der rheumatologischen Weiterbildung. Die Ergebnisse der hier vorgestellten Umfrage liefern wichtige Einblicke in die Situation der in Weiterbildung befindlichen Kolleginnen und Kollegen. An der Umfrage haben 102 Teilnehmer:innen aus dem gesamten Bundesgebiet teilgenommen. In der Umfrage zu den Weiterbildungsstellen 2022 wurden 478 Weiterbildungsstellen für Innere Medizin und Rheumatologie evaluiert, von denen, 82,8 % (*n* = 396) besetzt waren [14], sodass ca. 25 % der Weiterbildungsassistent:innen für Rheumatologie in Deutschland an der Umfrage teilgenommen haben.

Anzumerken ist, dass die Hälfte der Befragten an einer Universitätsklinik tätig war, sodass möglicherweise die Aussagen in Bezug auf die Ausbildungsstellen im nichtuniversitären Bereich unzureichend repräsentiert sind. Auf der anderen Seite weisen die Universitätskliniken die meisten klinischen Weiterbildungsstellen (45 %, 177 von 391 klinischen Weiterbildungsstellen) auf [12].

Zwei Drittel der Teilnehmer:innen waren weiblichen Geschlechts, und über 90 % der Teilnehmer:innen waren unter 40 Jahren. Drei Viertel der Teilnehmer:innen arbeiteten in Vollzeit, und ein Viertel ging einer Teilzeitbeschäftigung nach. Vergleichbare Ergebnisse konnten in den Umfragen der Deutschen Gesellschaft für Innere Medizin (Vollzeit 87 % und Teilzeit 13 %), der Deutschen Röntgengesellschaft (Vollzeit 83 % und Teilzeit 17 %), der Vereinigung von Assistenzärzten:innen in Weiterbildung zum Facharzt für Urologie (Vollzeit 90 % und Teilzeit 10 %) und der Gesellschaft für Gynäkologie und Geburtshilfe (Vollzeit 70 % und Teilzeit 30 %) ermittelt werden [2, 7, 9, 16].

Durch die Anonymität der Befragung ist von einem realen Bild der derzeitigen Situation von Assistenzärzt:innen in der rheumatologischen Weiterbildung auszugehen. Als positiv hervorzuheben ist, dass nahezu die Hälfte der Teilnehmer:innen (48,1 %) Zufriedenheit mit ihrer derzeitigen Arbeitssituation äußerte. Dies ist insbesondere vor dem Hintergrund einer höheren Zufriedenheit der Befragten im Vergleich zu den Ergebnissen der Inneren Medizin (38 %), Urologie (44 %) und der Gynäkologie (40 %) zu interpretieren [2, 7, 16]. Als Faktoren, welche die Arbeitssituation negativ beeinflussen, wurden neben der hohen Arbeitsbelastung v. a. der Anteil an arztfremden Tätigkeiten sowie der Einfluss auf das Privatleben genannt. So wurde den Themen zur Vereinbarkeit von Familie/Privatleben und der beruflichen Tätigkeit die höchste Priorität zugeordnet. Ein weiteres wichtiges Ergebnis der Befragung ist, dass Assistenzärzt:innen in der rheumatologischen Weiterbildung angaben, unter erheblichem Arbeitsdruck und Zeitmangel zu leiden. Da sich dies negativ auf ihre körperliche und geistige Gesundheit auswirken und zu Burn-out und anderen gesundheitlichen Problemen führen kann, scheint es wichtig, Maßnahmen zu ergreifen, um die „Work-Life-Balance“ zu verbessern und den Assistenzärzt:innen bessere Unterstützungssysteme zur Verfügung zu stellen.

Hervorzuheben ist die Angabe von mehr als drei Viertel der Teilnehmer:innen, dass an ihrem Weiterbildungsstandort kein strukturiertes Weiterbildungscurriculum mit von Beginn an vorgegebenen, planbaren Lerninhalten/Rotationen existiert. Hier liegen die Ergebnisse der aktuellen Umfrage ebenfalls auf dem Niveau der schon publizierten Umfrageergebnisse der Inneren Medizin (78 %), Radiologie (63 %), Urologie (70 %) und Gynäkologie (82 %) [2, 7, 9, 16]. Dies scheint ein Ansatzpunkt, der Optimierungsbedarf und -potenzial bietet. Dafür kann das Mustercurriculum der DGRh als eine Grundlage zur Implementierung einer standardisierten und strukturierten Aus- und Weiterbildung im Fachgebiet Innere Medizin und Rheumatologie in Deutschland dienen [13]. Hierbei ist die Implementierung eines Weiterbildungscurriculums mit einer erhöhten Zufriedenheit im Beruf vonseiten der Weiterbildungsassistent:innen assoziiert [16]. Die ergänzende Nutzung der Angebote der rheumatologischen Fortbildungsakademie kann die Situation der rheumatologischen Aus- und Weiterbildung in Kliniken und Praxis zusätzlich weiter verbessern.

Es waren 70 % der Befragten promoviert – mit einem deutlich höheren Ergebnis als in der Inneren Medizin (52 %), Radiologie (59 %), Urologie (44 %) und Gynäkologie (54 %) [2, 7, 9, 15]. Hierbei sind 55 % der Befragten wissenschaftlich tätig (Innere Medizin 19 %, Radiologie 51 %, Urologie 39 % und Gynäkologie 42 %) [2, 7, 9, 16] und arbeiten wissenschaftlich primär an klinisch orientierten Forschungsgebieten. Primär (72 %) wird die wissenschaftliche Arbeit in der Freizeit durchgeführt. Zur Stärkung der wissenschaftlichen Arbeit sollten Forschungszeiträume mit einer Freistellung von der klinischen Tätigkeit etabliert werden. Der hohe Anteil von wissenschaftlich tätigen Weiterbildungsassistent:innen muss allerdings vor dem Hintergrund einer relativen Überrepräsentation von Teilnehmer:innen aus universitären Einrichtungen eingeordnet werden.

In der Umfrage der AGJR 2019 wurde dargestellt, dass nur 19 % ein Mentoring erhalten [5]. In der aktuellen Umfrage gaben 40 % der Befragten an, ein Mentoring zu erhalten, sodass im Vergleich zu der Umfrage der AGJR eine Verbesserung der Situation zu vermerken ist. Nichtsdestotrotz sollte auch zukünftig eine weitere Verbesserung der Situation im Hinblick auf das Mentoring zur Durchführung der rheumatologischen Diagnostik (z. B. Gelenkpunktionen) erfolgen, um die Ausbildungsqualität zu verbessern.

Nach Abschluss der Facharztweiterbildung strebt die Hälfte der Befragten eine kombinierte stationäre und ambulante Tätigkeit an, die primär im angestellten Verhältnis durchgeführt werden soll. Hierbei sehen 74 % ihre Zukunft in einer Anstellung in einem medizinischen Versorgungszentrum, wobei hier die Ergebnisse zu einer Umfrage unter rheumatologischen Weiterbildungsassistent:innen von 2020 in Sachsen-Anhalt, Sachsen und Thüringen vergleichbar sind [10]. Die Übernahme einer selbstständig geführten rheumatologischen Praxis scheint dabei für viele keine erstrebenswerte berufliche Alternative darzustellen [10].

Entsprechend der Umfrage stellen in der beruflichen Tätigkeit die Vereinbarkeit von Familie und Beruf oder auch die „Work-Life-Balance“ sowie die Teilzeittätigkeit wichtige Faktoren dar. In der Umsetzung einer kombinierten stationären und ambulanten Tätigkeit und z. B. einer Teilzeitbeschäftigung ist die Anstellung in einem medizinischen Versorgungszentrum eine Option der beruflichen Betätigung. Des Weiteren bildet die ambulante spezialfachärztliche Versorgung eine zusätzliche Ergänzung für die rheumatologische Weiterbildung, da eine kombinierte stationäre und ambulante Betätigung möglich ist. Zusammenfassend sind hierbei für künftige Fachärzt:innen entsprechende berufliche Angebote zu schaffen, damit das Fachgebiet für Weiterbildungsassistent:innen attraktiv bleibt. Denn die Wahl des internistischen Schwerpunktes erfolgt oft bereits während des Medizinstudiums, und die mögliche Lebensführung in der entsprechenden Fachrichtung spielt eine entscheidende Rolle bei der Wahl der Fachrichtung [3, 8]. In diesem Zusammenhang ist auch das Fehlen eigenständiger rheumatologischer Lehrstühle bzw. rheumatologischer Abteilungen an jeder Universitätsklinik als kritisch zu bewerten, da die universitäre Präsenz eines Fachgebietes bei den Studierenden mit der Wahrnehmung des Fachgebietes als Weiterbildungsoption assoziiert ist [11, 17].

Zusätzlich sollte in regelmäßigen Intervallen eine solche Umfrage wiederholt werden, um die Auswirkung von Änderungen der Weiterbildung evaluieren und ggf. eine erneute Adaptation durchführen zu können.

## Schlussfolgerung

Zusammenfassend lassen sich aus den Ergebnissen der BEWUSST-Umfrage wichtige Erkenntnisse ziehen, die zu einer Anpassung und kontinuierlichen Optimierung der rheumatologischen Aus- und Weiterbildung in Deutschland unter Berücksichtigung der Sichtweise der in Weiterbildung befindlichen Kolleg:innen führen können. Eine Berücksichtigung der genannten negativen Implikationen ist vor dem Hintergrund des zunehmenden Mangels an internistischen Rheumatolog:innen und der steigenden Prävalenz entzündlich rheumatischer Erkrankungen dringend notwendig. Um eine weiterhin adäquate rheumatologische Versorgung zu gewährleisten, muss dringend die Zahl der Weiterbildungsstellen erhöht werden.

## Fazit für die Praxis

- Die Hälfte der teilnehmenden angehenden Rheumatolog:innen ist mit den Arbeitsbedingungen im Fachgebiet zufrieden.
- Das Mentoring sollte unter Nutzung des Mustercurriculums einen weiteren Ausbau mit Implementierung eines strukturierten Weiterbildungsprogrammes erfahren.
- Hinsichtlich der gewünschten kombinierten klinischen und ambulanten Tätigkeit sollten die vorhandenen Optionen (z. B. ambulant spezialärztliche Versorgung) ausgebaut werden bzw. neue berufliche Tätigkeitsfelder etabliert werden, damit das Fachgebiet auch für den Nachwuchs attraktiv bleibt.

### Supplementary Information





## Abkürzungen

AGJR: Arbeitsgemeinschaft Junge Rheumatologie
DGIM: Deutsche Gesellschaft für Innere Medizin
DGRh: Deutsche Gesellschaft für Rheumatologie
FA: Facharzt
VPN: Virtual Private Network
