# Supplementary material for: Survey on the working, training, and research conditions of resident physicians in internistic and rheumatological continuing education—BEWUSST
Source: Z Rheumatol. 2023 Aug 11;83(4):257–68. [Article in German] doi: 10.1007/s00393-023-01395-6 (PMC11058970; doi:10.1007/s00393-023-01395-6)
Supplement: Supplementary file 1 [file 393_2023_1395_MOESM1_ESM.pdf]

Befragung zu den Arbeits-, **W**eiterbildungs- und

Forsch**u**ngsbedingungen von

A**ss**istenzärztinnen und -ärzten in der internistisch-rheumatologischen

Wei**t**erbildung – **BEWUSST**

Themenbereiche der Umfrage:

	Themenbereich	Anzahl der Fragen
1.	Basisdaten	15
2.	Arbeitsbedingungen im Berufsalltag	5
3.	Ärztliche Fort- und Weiterbildung	17
4.	Vereinbarkeit von Beruf und Familie	10
5.	Vereinbarkeit von Arbeit und Forschung	16
6.	Perspektive Rheumatologe / Rheumatologin	14
7.	Praktische Tätigkeiten	22
8.	Persönliche Meinung / Kommentare	3

1 Basisdaten

Tabelle 1: Basischarakteristika der Umfrageteilnehmer.

**Teilnehmer**	Gesamt	n = 102
	Frauen	n = 68 (66,7 %)
	Männer	n = 34 (33,3 %)
	Divers	n = 0 (0 %)
**Alter**	20 – 24 Jahre	n = 0 (0 %)
	25 – 29 Jahre	n = 23 (22.5 %)
	30 – 34 Jahre	n = 41 (40,2 %)
	35 – 39 Jahre	n = 31 (30,4 %)
	40 – 44 Jahre	n = 3 (2,9 %)
	> 45 Jahre	n = 4 (3,9 %)
**Nationalität**	Deutsch	n = 94 (92,2 %)
	Andere	n = 8 (7,8 %)
**Anzahl der Kinder im Haushalt**	n = 1	n = 24 (23,5 %)
	n = 2	n = 39 (38,2 %)
	n = 3	n = 17 (16,7 %)
	n = 4	n = 17 (16,7 %)
	n = 5	n = 3 (2,9 %)
	n = 6	n = 0 (0 %)
	n > 6	n = 2 (2,0 %)
**Bundesland**	Baden-Württemberg	n = 18 (17,6 %)
	Bayern	n = 21 (20,6 %)
	Berlin	n = 7 (6,9 %)
	Brandenburg	n = 1 (1,0 %)
	Bremen	n = 0 (0 %)
	Hamburg	n = 5 (4,9 %)
	Hessen	n = 1 (1,0 %)
	Mecklenburg-Vorpommern	n = 0 (0 %)
	Niedersachsen	n = 6 (5,9 %)
	Nordrhein-Westfalen	n = 20 (19,6 %)
	Rheinland-Pfalz	n = 4 (3,9 %)
	Saarland	n = 0 (0 %)
	Sachsen	n = 6 (5,9 %)
	Sachsen-Anhalt	n = 4 (3,9 %)
	Schleswig-Holstein	n = 4 (3,9 %)
	Thüringen	n = 5 (4,9 %)

Tabelle 2: Facharztbezeichnung und Facharztausbildung.

**Facharztbezeichnung**	Facharztbezeichnung vorhanden<br>Keine Facharztbezeichnung vorhanden	n = 9 (8,8 %)<br>n = 93 (91,2 %)
**Welche Facharztbezeichnung streben sie derzeit an (1. Facharztbezeichnung)?**	Facharzt für Allgemeinmedizin - Mindestweiterbildungszeit 5 Jahre ?<br><br>Facharzt für Innere Medizin (gesamte Innere Medizin) - Mindestweiterbildungszeit 5 Jahre ?<br><br>Facharzt für Innere Medizin und Rheumatologie Mindestweiterbildungszeit 6 Jahre ?<br><br>Facharzt für Innere Medizin (gesamte Innere Medizin) und Facharzt für Innere Medizin und Rheumatologie - Mindestweiterbildungszeit 8 Jahre ?<br><br>Andere Facharztbezeichnung:	n = 9 (8,8 %)<br><br>n = 25 (24,5 %)<br><br>n = 59 (57,8 %)<br><br>n = 52 (51,0 %)<br><br>n = 7 (6,9 %)
**Was ist ihr Karriereziel (Mehrfachnennung möglich)?**	Facharzt im Krankenhaus<br>Oberarzt im Krankenhaus<br>Chefarzt im Krankenhaus<br>Facharzt in der Niederlassung<br>Akademische Laufbahn mit Habilitation/ Professur<br>Sonstiges	n = 23 (13,1 %)<br>n = 48 (27,3 %)<br>n = 6 (3,4 %)<br>n = 67 (38,1 %)<br>n = 29 (16,5 %)<br>n = 3 (1,7 %)
**Wo sind Sie derzeit primär tätig?**	Normalstation<br>Intensivstation/ ICU oder Rettungsstelle<br>Funktionsbereich (z.B. Sonographie etc.)<br>Tagesklinik<br>Klinikambulanz<br>Ambulante Sprechstunde einer rheumatologischen Praxis<br>Sonstiges (bitte angeben)	n = 47 (46,1 %)<br>n = 5 (4,9 %)<br>n = 1 (1,0 %)<br>n = 3 (2,9 %)<br>n = 34 (33,3 %)<br>n = 8 (7,8 %)<br>n = 4 (3,9 %)
**Für den Fall, dass Sie in einer Klinik tätig sind, in welcher Trägerschaft befindet sich das**	Das Krankenhaus, in dem ich arbeite, befindet sich in öffentlicher Trägerschaft (z.B. städtisches Krankenhaus und die meisten Universitätsklinika)	n = 73 (71,6 %)

**Krankenhaus, in dem Sie arbeiten?**	Das Krankenhaus, in dem ich arbeite, befindet sich in freigemeinnütziger Trägerschaft (durch Kirchen oder Wohlfahrtsverbände betriebene Krankenhäuser wie z.B. DRK-Kliniken)<br><br>Das Krankenhaus, in dem ich arbeite, befindet sich in privater Trägerschaft (z.B. Helios-, Rhön-,Asklepios-, Sana Kliniken usw.)	n = 18 (17,6 %)<br><br>n = 11 (10,8 %)
**Welcher Versorgungsstufe kann das Krankenhaus, in dem Sie arbeiten, am ehesten zugeordnet werden?**	Ich arbeite derzeit in einem Krankenhaus der Grund-, Regel- oder Schwerpunktversorgung<br><br>Ich arbeite derzeit in einem Krankenhaus der Maximalversorgung (kein Universitätsklinikum)<br><br>Ich arbeite derzeit in einem Universitätsklinikum<br><br>Andere bzw. weiß nicht	n = 36 (35,3 %)<br><br>n = 11 (10,8 %)<br><br>n = 48 (47,1 %)<br><br>n = 7 (6,9 %)

Tabelle 3: Mentoring im Rahmen der Facharztweiterbildung.

**Haben Sie einen Mentor, der Sie betreut?**	Ja	n = 46 (45,1 %)
	Nein	n = 39 (38,2 %)
	Ab und zu	n = 17 (16,7 %)
**Finden regelmäßig Feedbackgespräche mit Ihren Vorgesetzten statt?**	Ja	n = 40 (39,2 %)
	Nein	n = 36 (35,3 %)
	Ab und zu	n = 26 (25,5 %)

2. Arbeitsbedingungen im Berufsalltag

Tabelle 4: Arbeitsbedingungen im Rahmen der Facharztweiterbildung.

**Wie zufrieden sind Sie insgesamt mit Ihrer augenblicklichen beruflichen Situation?**	Sehr unzufrieden	n = 10 (9,8 %)
	Eher unzufrieden	n = 17 (16,7 %)
	Unentschieden – teils, teils	n = 26 (25,5 %)
	Eher zufrieden	n = 37 (36,3 %)
	Sehr zufrieden	n = 12 (11,8 %)
**Welche Faktoren sorgen bei Ihnen für Unzufriedenheit? (bis zu drei Antworten möglich)**	Hohe zeitliche Arbeitsbelastung	n = 41 (14,6 %)
	Unregelmäßige Arbeitszeiten und Schichtdient.	n = 13 (4,6 %)
	Hoher Arbeitsverdichtung	n = 26 (9,3 %)
	Schlechte Vereinbarkeit von Familie und Beruf	n = 13 (4,6 %)
	Keine Zeit für Forschung	n = 17 (6,1 %)
	Mein Privatleben leidet	n = 21 (7,5 %)
	Keine eigenständigen medizinischen Entscheidungen möglich	n = 2 (0,7 %)
	Starker ökonomischer Einfluss auf medizinische Entscheidungen	n = 16 (5,7 %)
	Schlechtes Arbeitsklima	n = 5 (1,8 %)
	Starre Hierarchien	n = 4 (1,4 %)
	Hoher Anteil arztfremder Tätigkeiten	n = 35 (12,5 %)
	Mangelnde Qualität der medizinischen Weiterbildung	n = 13 (4,6 %)
	Fehlende Anleitung und Supervision	n = 19 (6,8 %)
	Geringe Anerkennung für die geleistete Arbeit	n = 13 (4,6 %)
	Schlechte Organisation der Abläufe/ schlechte Absprachen	n = 26 (9,3 %)
	Mangelnde Zukunftsperspektive	n = 269 (3,2 %)
	Sonstiges: Freitext	n = 7 (2,5 %)
**Bitte schätzen Sie den Anteil folgender Tätigkeiten an Ihrem beruflichen Arbeitsalltag im Hinblick auf patientenorientiertes**	Angaben jeweils in Prozent mit einer Gesamtsumme von 100%.	
	Anteil von Arbeiten mit und am Patienten (z.B. Patientenaufnahme, Visite, Patienten-/Angehörigengespräche, technische	42,7 %

**Arbeiten ein (in Prozent; die Gesamtsumme soll 100 Prozent ergeben).**	Untersuchungen am Patienten wie Sonographie etc.)<br><br>Anteil patientenbezogener Arbeiten (z.B. Tumorkonferenz, Fallbesprechung mit der Oberärztin/-arzt, Dokumentation von Verläufen, Arztbriefe)<br><br>Anteil nicht ärztlicher bzw. patientenferner Tätigkeiten (z.B. Befunde oder Termine organisieren, Untersuchungen anmelden, Diagnosen oder Eingriffe kodieren)	 <br><br>31,8 %<br><br><br><br><br>25,5 %
**Wie hat sich die Qualität der Patientenversorgung Ihrer Meinung nach über die letzten Jahre verändert?**	(Bitte geben Sie die Veränderung als Wert auf einer Skala zwischen -5/sehr negativ über 0/gar nicht bis +5/sehr positiv an)<br><br>-5 (sehr negativ)<br>-4<br>-3<br>-2<br>-1<br>0 (gar nicht)<br>1<br>2<br>3<br>4<br>5 (sehr positiv)	 <br><br><br>n = 2 (2,0 %)<br>n = 6 (5,9 %)<br>n = 15 (14,7 %)<br>n = 22 (21,6 %)<br>n = 18 (17,6 %)<br>n = 16 (15,7 %)<br>n = 9 (8,8 %)<br>n = 6 (5,9 %)<br>n = 6 (5,9 %)<br>n = 0 (0 %)<br>n = 2 (2,0 %)
**Haben Sie bereits aus Unzufriedenheit über Ihre Arbeitsbedingungen eine oder mehrere der nachfolgend genannten Konsequenzen gezogen bzw. daran gedacht?**	***Die Arbeitszeit reduziert***<br>Nur daran gedacht<br>Getan<br>Nicht zutreffend	 n = 51 (50,0 %)<br>n = 21 (20,6 %)<br>n = 30 (29,4 %)
	Den Arbeitsplatz (Abteilung oder Krankenhaus) gewechselt<br>Nur daran gedacht<br>Getan<br>Nicht zutreffend	 <br>n = 45 (44,1 %)<br>n = 34 (33,3 %)<br>n = 23 (22,5 %)
	Die praktisch ärztliche Tätigkeit aufzugeben<br>Nur daran gedacht<br>Getan	 <br>n = 39 (38,2 %)<br>n = 2 (2,0 %)

	Nicht zutreffend	n = 61 (59,8 %)
	Wechsel der Facharztrichtung	
	Nur daran gedacht	n = 31 (30,4 %)
	Getan	n = 12 (11,8 %)
	Nicht zutreffend	n = 59 (57,8 %)
	Wechsel ins Ausland	
	Nur daran gedacht	n = 33 (32,4 %)
	Getan	n = 0 (0 %)
	Nicht zutreffend	n = 69 (67,6 %)

3 Ärztliche Fort- und Weiterbildung

Tabelle 5: Arbeitszeiten.

**Arbeiten Sie derzeit in Vollzeit oder in Teilzeit?**	Vollzeit	n = 77 (75,5 %)
	Teilzeit	n = 25 (24,5 %)
**Wenn Sie in Teilzeit arbeiten, fühlen Sie sich hierdurch im Hinblick auf das Fortkommen in Ihrer Weiterbildung benachteiligt?**	Nein, ich fühle mich dadurch nicht benachteiligt	n = 10 (40,0 %)
	Ja, ich fühle mich dadurch benachteiligt aus folgendem Grund/Gründen	n = 15 (60,0 %)

Tabelle 6: Weiterbildungsermächtigung und Arbeitsvertrag.

**Liegt die volle Weiterbildungsermächtigung für den Facharzt für Innere Medizin und Rheumatologie an Ihrer Einrichtung vor?**	Nein<br><br>Ja	n = 19 (18,6 %)<br><br>n = 83 (81,4 %)
**Haben Sie mit Antritt der ersten Anstellung einen Arbeitsvertrag über die gesamte Weiterbildungszeit erhalten?**	Nein<br><br>Ja	n = 65 (63,7 %)<br><br>n = 37 (36,3 %)
**Sollten Sie befristete Arbeitsverträge erhalten haben, welche Zeitdauer wies der kürzeste Arbeitsvertrag auf?**	3 Monate oder kürzer	n = 4 (6,2 %)
	6 Monate	n = 4 (6,2 %)
	1 Jahr	n = 18 (27,7 %)
	2 Jahre	n = 28 (43,1 %)
	3 Jahre	n = 9 (13,8 %)
	4 Jahre	n = 1 (1,5 %)
	5 Jahre	n = 1 (1,5 %)
	6 Jahre	n = 0 (0 %)
	7 Jahre	n = 0 (0 %)
	8 Jahre	n = 0 (0 %)
**Wie viele befriste Arbeitsverträge haben Sie bislang erhalten?**	1	n = 20 (19,6 %)
	2	n = 36 (35,3 %)
	3	n = 26 (25,5 %)
	4	n = 9 (8,8 %)
	5	n = 3 (2,9 %)
	≥6	n = 8 (7,8 %)

Tabelle 7: Weiterbildung.

**Gibt es bei Ihnen ein strukturiertes Weiterbildungscurriculum mit von Beginn an fest geplanten Lerninhalten/ Rotationen?**	Nein<br><br>Ja	n = 79 (77,5 %)<br><br>n = 23 (22,5 %)
**Gibt es einen Vertreter der Weiterbildungsassistenten und Weiterbildungsassistentinnen in der Leitung des Departments oder der Klinik?**	Nein<br><br>Ja	n = 43 (42,2 %)<br><br>n = 59 (57,8 %)
**Werden die Rotationen transparent von Seiten der Klinikleitung dargestellt?**	Nein<br><br>Ja	n = 59 (57,8 %)<br><br>n = 43 (42,2 %)
**Werden die Rotationen durch die Klinikleitung mit den Weiterbildungsassistenten und Weiterbildungsassistentinnen diskutiert?**	Nein<br><br>Ja	n = 44 (43,1 %)<br><br>n = 58 (56,9 %)
**Wie viele Rotationen gibt es im Rahmen der Facharztweiterbildung?**	0<br>1<br>2<br>3<br>4<br>5<br>6<br>7<br>>7	n = 7 (6,9 %)<br>n = 8 (7,8 %)<br>n = 17 (16,7 %)<br>n = 24 (23,5 %)<br>n = 25 (24,5 %)<br>n = 10 (9,8 %)<br>n = 1 (1,0 %)<br>n = 2 (2,0 %)<br>n = 8 (7,8 %)
**Werden Sie Ihre Weiterbildung in der vorgesehen Regelweiterbildungszeit abschließen können (z.B. 5**	Nein<br><br>Ja	n = 32 (31,4 %)<br><br>n = 70 (68,6 %)

**Jahre für den FA Innere Medizin)?**		
**Meinen Sie, dass Sie die nach Logbuch geforderten Weiterbildungsinhalte in der Regelweiterbildungszeit erlernt haben werden?**	Nein<br><br>Ja	n = 61 (59,8 %)<br><br>n = 41 (40,2 %)
**In welcher Form findet an Ihrer Klinik das jährlich geforderte Weiterbildungsgespräch statt?**	Als überwiegend strukturiertes und konstruktives Gespräch, das mir hilft<br><br>Als beiläufiges Gespräch, dass eher dem Zweck der Dokumentation im Logbuch dient<br><br>Gar nicht	n = 38 (37,3 %)<br><br>n = 42 (41,2 %)<br><br>n = 22 (21,6 %)
**Meinen Sie, dass die Inanspruchnahme externer Fortbildungen notwendig ist, um die Kompetenzen laut Weiterbildungsordnung bis zum Ende der Weiterbildungszeit zu erlernen?**	Nein, ich denke nicht, dass dafür externe Weiterbildungen/ Fortbildungen in Anspruch genommen werden müssen<br><br>Ja, ich glaube die Inanspruchnahme externer Angebote ist hilfreich<br><br>Ja, ich glaube die Inanspruchnahme externer Angebote ist unerlässlich	n = 4 (3,9 %)<br><br>n = 57 (55,9 %)<br><br>n = 41 (40,2 %)
**Wie oft finden Interne Weiterbildungen statt?**	Mehrmals wöchentlich<br>1x wöchentlich<br>1x monatlich<br>Alle 1-2 Monate<br>Gar nicht	n = 14 (13,7 %)<br>n = 45 (44,1 %)<br>n = 20 (19,6 %)<br>n = 10 (9,8 %)<br>n = 13 (12,7 %)

4 Vereinbarkeit von Beruf und Familie

Tabelle 8: Familie und Beruf.

**Die folgenden Fragen zu Vereinbarkeit von Beruf und Familie richten sich in erster Linie an Ärztinnen und Ärzte mit Kind/Kindern. Aber auch wenn Sie kein Kind/Kinder haben, können Sie die nachfolgenden Fragen sehr gerne beantworten.**	Ich habe ein Kind/Kinder<br>Ich habe kein Kind/Kinder, möchte die Fragen zu Beruf und Familie dennoch beantworten<br>Ich habe kein Kind/Kinder und möchte die Fragen zu Familie und Beruf überspringen	n = 37 (36,3 %)<br>n = 35 (34,3 %)<br>n = 30 (29,4 %)
**Im Folgenden nur Beantwortung der Fragen, wenn Kind/Kinder vorhanden bzw. die Fragen dennoch beantworten wollen.**		
**Ich bin zufrieden mit der Vereinbarkeit von Familie und Beruf an meinem Arbeitsplatz.**	trifft voll zu<br>trifft eher zu<br>teils, teils<br>trifft eher nicht zu<br>trifft nicht zu	n = 5 (6,9 %)<br>n = 20 (27,8 %)<br>n = 21 (29,2 %)<br>n = 16 (22,2 %)<br>n = 10 (13,9 %)
**Es gibt regelmäßig Situationen, in denen ich familiäre Aufgaben zu Gunsten der Arbeit vernachlässigen muss oder umgekehrt.**	trifft voll zu<br>trifft eher zu<br>teils, teils<br>trifft eher nicht zu<br>trifft nicht zu	n = 17 (23,6 %)<br>n = 29 (40,3 %)<br>n = 17 (23,6 %)<br>n = 6 (8,3 %)<br>n = 3 (4,2 %)
**Ich fühle mich bei der Vereinbarkeit von Beruf und Familie von meinem Arbeitgeber unterstützt (z.B. durch flexible Gestaltung der Arbeitszeiten oder Dienstpläne).**	trifft voll zu<br>trifft eher zu<br>teils, teils<br>trifft eher nicht zu<br>trifft nicht zu	n = 10 (13,9 %)<br>n = 11 (15,3 %)<br>n = 19 (26,4 %)<br>n = 20 (27,8 %)<br>n = 12 (16,7 %)
**Ich fühle mich bei der Vereinbarkeit von Beruf und Familie von meinen Kollegen unterstützt (z.B. durch flexible Gestaltung der Arbeitszeiten).**	trifft voll zu<br>trifft eher zu<br>teils, teils<br>trifft eher nicht zu<br>trifft nicht zu	n = 9 (12,5 %)<br>n = 26 (36,1 %)<br>n = 21 (29,2 %)<br>n = 11 (15,3 %)<br>n = 5 (6,9 %)

**Welche der folgenden Faktoren würden Ihnen eine gute Balance zwischen Arbeit und einem Privatleben mit familiären Aufgaben erleichtern/ermöglichen? (Auswahl der drei wichtigsten Faktoren)**	Flexiblere Gestaltung der Arbeitszeiten z.B. durch mehr Mitsprache bei der Festlegung der Arbeitszeit<br><br>Besser planbare oder regelmäßigere Arbeitszeiten<br><br>Weniger Überstunden<br><br>Kinderbetreuungsplatz<br><br>Ganztagsbetreuung<br><br>Kinderbetreuungsplatz in der Nähe der Arbeitsstelle<br><br>Mehr Rücksicht von Kolleginnen und Kollegen<br><br>Teile der Arbeit zu Hause erledigen (Dokumentation, Arztbriefe über Intranet/VPN)<br><br>Optionaler Betreuungsdienst für Kinder bzw. Angehörige in Notfällen, bei Sitzungen oder während der Schulferien.<br><br>Finanzielle Unterstützung<br><br>Mentorenprogramm<br><br>Ich bin mit dem derzeitigen Angebot zufrieden<br><br>Sonstiges: Freitext	n = 43 (21,4 %)<br><br>n = 29 (14,4 %)<br><br>n = 39 (19,4 %)<br><br>n = 9 (4,5 %)<br><br>n = 8 (4,0 %)<br><br>n = 4 (2,0 %)<br><br>n = 2 (1,0 %)<br><br>n = 40 (19,9 %)<br><br>n = 9 (4,5 %)<br><br>n = 4 (2,0 %)<br><br>n = 5 (2,5 %)<br><br>n = 6 (3,0 %)<br><br>n = 3 (1,5 %)
**Familienfreundlichkeit wird an meinem Arbeitsplatz bspw. in Fortbildungsangeboten, Newslettern sowie**	trifft voll zu<br>trifft eher zu<br>teils zu<br>trifft eher nicht zu<br>trifft nicht zu	n = 4 (5,6 %)<br>n = 9 (12,5 %)<br>n = 12 (16,7 %)<br>n = 28 (38,9 %)<br>n = 19 (26,4 %)

**Unterstützungsangeboten thematisiert und gehört somit zur Unternehmenskultur.**		
**Ich habe mich bisher gegen mehr familiäre Verantwortung entschieden, weil dies mit meinem beruflichen Werdegang nicht vereinbar war.**	trifft voll zu<br>trifft eher zu<br>teils zu<br>trifft eher nicht zu<br>trifft nicht zu	n = 7 (9,7 %)<br>n = 17 (23,6 %)<br>n = 6 (8,3 %)<br>n = 16 (22,2 %)<br>n = 26 (36,1 %)
**Rücksicht auf Mitarbeiter mit familiären Verpflichtungen geht häufig zu Lasten von Mitarbeitern ohne familiäre Verpflichtungen.**	trifft voll zu<br>trifft eher zu<br>teils zu<br>trifft eher nicht zu<br>trifft nicht zu	n = 29 (40,3 %)<br>n = 20 (27,8 %)<br>n = 16 (22,2 %)<br>n = 4 (5,6 %)<br>n = 3 (4,2 %)
**Haben Sie Elternzeit genommen?**	Ich habe bisher keine Elternzeit genommen<br><br>Ich habe bereits Elternzeit genommen	n = 41 (56,9 %)<br><br>n = 31 (43,1 %)
**Wenn Sie bereits eine Elternzeit genommen haben, ergänzen Sie bitte die maximale Dauer in vollen Monaten**	Kind 1<br>Kind 2<br>Kind 3<br>Kind 4	58,5 Monate<br>20,1 Monate<br>10,4 Monate<br>11,0 Monate

5 Vereinbarkeit von klinischem Arbeitsalltag mit Forschung und Wissenschaft

Tabelle 9: Wissenschaftliche Tätigkeit.

**Sind Sie promoviert?**	Nein, ich bin nicht promoviert und strebe dies auch nicht an.	n = 10 (9,8 %)
	Nein, ich bin noch nicht promoviert, plane aber eine bzw. arbeite derzeit an einer Dissertation.	n = 21 (20,6 %)
	Ja, ich bin promoviert.	n = 71 (69,6 %)
**Sind Sie derzeit wissenschaftlich tätig oder wollen dies in Zukunft sein?**	Nein	n = 46 (45,1 %)
	Ja	n = 56 (54,9 %)
**Mit welchem wissenschaftlichen Gebiet befasst sich Ihre Forschungsarbeit? (Mehrfachnennung möglich)**	Experimentell (Grundlagenforschung)	n = 21 (18,3 %)
	Klinisch orientiert.	n = 84 (73,0 %)
	Epidemiologisch.	n = 10 (8,7 %)
**Wann führen Sie Ihre wissenschaftliche Arbeit durch?**	Während der Arbeitszeit, neben der klinischen Tätigkeit.	n = 27 (26,5 %)
	In der Freizeit im Anschluss an die klinische Tätigkeit.	n = 73 (71,6 %)
	Es besteht eine Freistellung zur Durchführung der Forschungsarbeit.	n = 2 (2,0 %)
**Wird Ihnen die Teilnahme an wissenschaftlichen Kongressen ermöglicht?**	Nein	n = 18 (17,6 %)
	Ja	n = 84 (82,4 %)
**Wenn ja, folgende Frage beantworten**		
**An welchen wissenschaftlichen Kongressen nehmen Sie jährlich teil?**	National (z. B. Wissenschaftlicher Jahreskongress der Deutschen Gesellschaft für Rheumatologie)	n = 0 (0 %)
		n = 0 (0 %)

	International (z. B. Annual European Congress of Rheumatology)	
**Auf wieviel wissenschaftlichen Kongressbeiträgen jährlich sind Sie ein Koautor?**	Mittelwert ± Standardabweichung	1,2 ± 0,7
**Auf wieviel wissenschaftlichen Kongressbeiträgen jährlich sind Sie ein Erstautor?**	Mittelwert ± Standardabweichung	0,4 ± 0,5
**Streben Sie eine Habilitation an?**	Nein<br><br>Ja	n = 73 (71,6 %)<br><br>n = 29 (28,4 %)
**Wenn Sie eine Habilitation anstreben, in welchen Zeitraum ist mit einer Fertigstellung zu rechnen (in Jahren)?**	Mittelwert ± Standardabweichung	5,3 ± 3,0 Jahre<br>(Range: 0,5 – 10 Jahre)

Tabelle 10: Drittmittelprojekte.

**Sind Sie mit Antragsteller / Mitantragsteller von Projektanträgen?**	Industriemittel.	n = 5 (4,9 %)
	Öffentliche Mittel (z. B. Deutsche Forschungsgemeinschaft).	n = 11 (10,8 %)
	Nein.	n = 86 (84,3 %)

Tabelle 11: Lehrtätigkeit.

**Sind Sie an der studentischen Lehre beteiligt?**	Nein	n = 39 (38,2 %)
	Ja	n = 63 (61,8 %)
**Wenn ja, welche Lehrveranstaltungen werden von Ihnen durchgeführt/mitgestaltet?**	Vorlesungen	n = 3 (2,3 %)
	Seminare	n = 29 (22,5 %)
	Praktika	n = 37 (28,7 %)
	Qualifizierter Unterricht am Krankenbett	n = 47 (36,4 %)
	POL-Kurse (Problemorientiertes Lernen)	n = 13 (10,1 %)

6 Perspektive Rheumatologin / Rheumatologe

Tabelle 12: Tätigkeit als Rheumatologin / Rheumatologe.

**Ist eine Facharztausbildung im ambulanten Bereich von Interesse?**	Nein<br><br>Ja	n = 23 (22,5 %)<br><br>n = 79 (77,5 %)
**Wurden bereits Ausbildungsabschnitte im niedergelassenen Bereich absolviert?**	Nein<br><br>Ja	n = 88 (86,3 %)<br><br>n = 14 (13,7 %)
**Wird von Ihnen später eine Teilzeittätigkeit als Facharzt angestrebt?**	Nein<br><br>Ja	n = 46 (45,1 %)<br><br>n = 56 (54,9 %)
**Streben Sie eine klinische oder ambulante Tätigkeit an?**	Klinik<br><br>Ambulante Tätigkeit<br><br>Klinik und ambulante Tätigkeit	n = 13 (12,7 %)<br><br>n = 35 (34,3 %)<br><br>n = 54 (52,9 %)
**Soll die ambulante Tätigkeit in der selbstständigen Niederlassung oder als angestellter Arzt/Ärztin (z. B. in einem Medizinischen Versorgungszentrum) durchgeführt werden?**	Selbstständig<br><br>Angestellt	n = 32 (36,0 %)<br><br>n = 57 (64,0 %)
**Wie schätzen Sie die Wahrscheinlichkeit Ihrer Niederlassung als Rheumatologe ein (Angabe in Prozent)?**		48 ± 47 %
**Ist eine Tätigkeit in einem Medizinischen Versorgungszentrum als angestellter Arzt eine Option?**	Nein<br><br>Ja	n = 26 (25,5 %)<br><br>n = 76 (74,5 %)
**Besteht ein Kontakt zu einem niedergelassenen Rheumatologen in Bezug auf eine potenzielle**	Nein<br><br>Ja	n = 84 (82,4 %)<br><br>n = 18 (17,6 %)

**Praxisübernahme / Praxisteilhaber?**		
**Ist eine Hospitation in einer Praxis für Sie interessant?**	Nein<br><br>Ja	n = 21 (20,6 %)<br><br>n = 81 (79,4 %)
**Bitte geben Sie die für Sie wichtigsten Argumente *für* eine Kliniktätigkeit (als RheumatologIn) an?**	Interessante Fälle<br><br>Spannende Fälle, akute Rheumatologie<br><br>Forschung seltenere Krankheiten<br><br>Kontakt zur Community, Expertenstatus, innovativeres Umfeld<br><br>Forschungsmöglichkeit Austausch mit Kollegen leichter möglich Interdisziplinäre Fälle<br><br>mehr möglichkeit arbeit/verantwortung abzugeben<br><br>Arbeitsbedingungen + Gehalt<br><br>Teamarbeit, interdisziplinäres Arbeiten, Lehre, komplexe Krankheitsfälle<br><br>Kränkere Patienten, Fallbesprechungen, Röntgenbesprechung<br><br>Komplexere Fälle, interdisziplinärer Austausch, Verantwortungsteilung, engere Zusammenarbeit mit Kolleg:innen, mehr studentische Lehre<br><br>Interdisziplinäres arbeiten Möglichkeiten zur Zeit nähen Diagnostik Fachwissen von OberäztInnen/ChafärztInnen<br><br>interessantere Fälle, mehr diagnostische Moöglichkeiten<br><br>abwechslungsreiche Tätigkeit, vielfältige Krankheitsbilder, Lehrtätigkeit, Teamarbeit<br><br>Man kann deutlich mehr viel lernen.<br><br>interdisziplinäre Zusammenarbeit mit anderen Fachabteilungen, Möglichkeit der wissenschaftlichen Arbeit, schnelle Diagnostik<br><br>Breites Spektrum, mehr Zeit für die Patientin, wissenschaftlicher Austausch<br><br>Breites Spektrum an patienten, eigenständige Durchführung der Diagnostik und damit die Möglichkeit den Fall komplett zu verstehen	

	kollegialer Austausch<br><br>Interdisziplinäre Tätigkeit<br><br>Großes Team<br><br>Breitere Möglichkeiten der Diagnostik, Arbeit im Team mit Möglichkeit zum Austausch, 'spannendere' Krankheiten bzw. Krankheitsphasen<br><br>Versorgung von Notfällen, breites Diagnostikspektrum direkt vor Ort, interdisziplinäre Zusammenarbeit<br><br>Erweitertes Spektrum der Diagnostik Komplexere Patientenfälle Kollegium<br><br>Spannende, komplizierte und komplexe Fälle. Behandlung schwerer Verläufe. Weiterhin breite internistische Aufarbeitung.<br><br>Komplexere Fälle, Möglichkeit der Rehabilitation zu begleiten von Patienten. Austausch mit Kollegen.<br><br>Bessere Ressourcen, weniger Finanzverantwortung, einfacher Urlaub<br><br>Flxiblere Arbeitszeiten, keine Nacht/WE dienste, besserer Verdienst.<br><br>Spannende Krankheitsbilder, interdisziplinäre Zusammenarbeit<br><br>Team<br><br>Interdisziplinäres Arbeiten, viel kollegialer Austausch, komplexe Fälle, Forschung, persönliche fachliche Weiterentwicklung<br><br>interdisziplinärer<br><br>Interdisziplinäre Konferenzen<br><br>Mehr Zeit für PatientInnen, mehr Zeit für Forschung<br><br>Krankheitsspektrum, Planbarkeit<br><br>Forschung<br><br>Interdisziplinäres Arbeiten, komplexe Fälle<br><br>kein eigenes finanzielles Risiko durch die Niederlassung und somit Konzentration auf gesundheitliche Versorgung der Patienten und nicht gebunden an ökonomische Vorgaben

	Austausch mit fachlichen Kollegen verschiedener Richtungen, Nähe zu neuen wissenschaftlichen Erkenntnissen, anspruchsvollere Fälle<br><br>Angestelltenverhältnis, neuste Therapie, Diagnostische Möglichkeiten, Forschung<br><br>Teamarbeit, kollegiale Unterstützung, radiologische Besprechungen<br><br>Hohe klinische Variabilität der Krankheitsbilder, interkollegialer Austausch<br><br>Abwechslungsreichtum, Arbeit im Team<br><br>Interessante Fälle<br><br>Gute und schnell Verfügbarkeit von Untersuchungsmöglichkeiten, interdisziplinäres Arbeiten.<br><br>Spannende Krankheitsbilder Angestellten Verhältnis<br><br>Diagnostische und therapeutische Mittel, Netzwerk<br><br>Fortführung Forschungstätigkeit formal geregelte Arbeitszeiten (real jedoch eher nicht) komplexe Versorgung möglich geringes persönliches Risiko<br><br>Vielfalt der Erkrankungen und Komplexität<br><br>Komplexität<br><br>Schwerere Krankheitsverläufe, seltenere Erkrankungen<br><br>Spannende Fälle, abwechslungsreiche Tätigkeit, Forschung<br><br>Spannender und schwerere Fälle, mehr Diagnostik<br><br>Spätere Ausbildung von Nachwuchs<br><br>Interessante Fälle ;)<br><br>Diagnostikverfügbarkeit, Akutmedizin, interdisziplinärer Austausch<br><br>Viele spannende Fälle, viel Abwechslung, mehr Diagnostik möglich<br><br>Mehr Zeit für Diagnostsik und den einzelnen Patienten<br><br>- interessante und seltenere Krankheisbilder - schnellere Möglichkeit weiterführender Untersuchungen (z.B. MRT, Biopsien, etc.)

	Schwerere Krankheitsbilder, akute Probleme, interessant, komplettes Spektrum, komplexe Fälle<br><br>spannend<br><br>Schnellere diagnostische Möglichkeiten<br><br>Spannend<br><br>nur die Forschung für mich<br><br>Das Patientenkollektiv (Vaskulitiden, Kollagenosen)<br><br>Differentialdiagnostik ermöglichen, schnelle Hilfestellung bei organbeteiligung, schwere Verläufe beherrschen<br><br>Sicherheit des Arbeitsplatzes, Teamarbeit und Rücksprache, interdisziplinäre Versorgung von Patienten<br><br>In Universitätsambulanz gäbe es mehr Mittel, Studierendenunterricht könnte weitergegeben werden<br><br>Arbeiten im Team, Möglichkeit der unkomplizierten Rücksprache, interdisziplinäres Arbeiten<br><br>Interessante medizinische Fälle<br><br>Einfache Interdisziplinarität<br><br>Interessante Fälle<br><br>Austausch mit Kolleginnen<br><br>Mehr Zeit und mehr Flexibilität<br><br>Ausbildung Nachwuchs seltene Fälle<br><br>Interessante Fälle und Vielfalt an diagnostischen und therapeutischen Möglichkeiten<br><br>Komplexe spannende Fälle, Diskussion und Austausch mit Kollegen<br><br>Komplexere Krankheitsbilder, bessere Möglichkeiten zur Funktionsdiagnostik, kollegiale Zusammenarbeit<br><br>Spannende Fälle, Interdisziplinarität

	Interessante Fälle, Kollegium<br><br>Mehr Kontakt mit Kollegen (auch aus andere Fachrichtungen), Interessante Fälle, mehr Abwechslung<br><br>Interessantere Fälle, Möglichkeit zur Forschung<br><br>Ich sehe für mich persönlich keine Argumente als Rheumatologin mittel- oder langfristig in der Klinik tätig zu sein, da ich trotz meiner Tätigkeit in unserer ASV Ambulanz immer werde Dienste machen müssen<br><br>Finanzielle Sicherheit<br><br>Aktueller Wissensstand, Fallkonplexität, Akutversorgung, Maximalversorgung<br><br>Maximalversorgung, spannende Fälle<br><br>Komplizierte und interessante Fälle<br><br>Arbeit im Team mit fächerübergreifendem Austausch, Patientenspektrum, Therapie- und Diagnostikmöglichkeiten<br><br>Interdisziplinäre Arbeit, komplexe Fälle, Akutmedizin<br><br>Interessantere Fälle, multidisziplinär arbeiten, Studentenunterricht<br><br>Forschung u spannende Fälle<br><br>Interdisziplinäre Arbeit<br><br>Akut Medizin<br><br>Sehr seltene Krankheitsbilder werden geschult, viele Therapieoptionen<br><br>Austausch mit Kollegen, fachübergreifende Versorgung<br><br>Teamarbeit, Kollegen<br><br>Weniger Probleme mit Kostenübernahmen, bessere Zeitplanung.<br><br>Weniger Organisationstätigkeit.
**Bitte geben Sie die für Sie wichtigsten Argumente für *gegen* eine Kliniktätigkeit (als RheumatologIn) an?**	Arbeitszeiten<br><br>Überstunden, keine Anerkennung, zu viel Arbeit, Respektlosigkeit des Arbeitgebers

	Unflexible Arbeitszeiten<br><br>Arbeitsbelastung<br><br>Arbeitszeiten Dienste<br><br>Fremdbestimmung<br><br>Schlechtes Arbeitsklima<br><br>Hohe Arbeitsbelastung, schlechtere Work Life Balance<br><br>Verdienst, wenig Gestaltungsmöglichkeiten, Hierarchien<br><br>Sehr schlechte Arbeitsabläufe, wenig Gestaltungsfreiheit, vermehrte Abhängigkeiten, Wochenend- oder Hintergrunddienste, seltener Teilzeit<br><br>Wochenendarbeit Nachtarbeit<br><br>Immer wieder Schichtdienst in der Notaufnahme, keine geregelten Arbeitszeiten, viele Überstunden, schlechte ineffiziente Strukturen<br><br>Überstunden, schlechtere Planbarkeit, schlechte Organisationsstrukturen<br><br>Zu viel Arbeit. Überstunden.<br><br>schlechte Arbeitsbedingungen, hohe Arbeitsbelastung, wenig Entscheidungsspielraum, schlechte Vereinbarkeit von Familie und Beruf, schlechte Stimmung<br><br>Keine größeren Einflussmöglichekeiten auf Arbeitszeiten und Einkommen<br><br>Hohe Arbeitsbelastung, tägliche Überstunden , keine Chance zum Aufstieg in der Abteilung oder Tätigkeit in der ASV<br><br>unterbewertet<br><br>Arbeitszeitbelastung<br><br>Arbeitszeiten<br><br>Dienste, deutlich geringeres Gehalt als ambulant, Karriere via Wissenschaft mit vielen Kindern kaum möglich<br><br>Schichtdienst, Wochenenddienste, Überstunden, Vergütung

	Dienstbelastung Eingeschränkte Selbständigkeit<br><br>Wenig FA oder OÄ-Stellen<br><br>Überstunden, Finanzierungspläne der Kliniken, schlechtere bezahlung<br><br>Dienste, Verdienst, Gestaltungsfreiheit<br><br>WE/Nachtdienste, zu viele Nebentätigkeiten.<br><br>Dienstbelastung, Überstunden<br><br>Zeit<br><br>Dienste, Arbeitsbelastung, ggf. fehlende Aufstiegschancen, ggf. schlechtere Vergütung<br><br>schichtdienst, forschung, gehalt<br><br>Rückschrittliche IT-Infrastruktur, Datenschutz steht über Patientenversorgung<br><br>Nacht- und Wochenenddienste; schlechte Organisationstrukturen<br><br>Niedriges Gehalt<br><br>Arbeitsbelastung<br><br>Schichtdienst, schlechte Planbarkeit, schlechte Vereinbarkeit mit der Familie<br><br>Arbeit im Schichtdienst (aktuell 24Sunden-Dienste mit schlechter Bezahlung) zusätzlich zur 40-Stunden-Wochen<br><br>24h Dienste, Unflexibilität der Arbeitszeiten<br><br>Arbeitspensum<br><br>Arbeitsverdichtung<br><br>Unvorhergesehene Arbeitsbelastung (Notfälle, Ausfälle bei Kollegen, Dienstübernahmen), 24-h-Dienste<br><br>Forschungsdruck, Arbeitsbelastung<br><br>Dienstbelastung<br><br>Dienstsystem, Höhe Arbeitsbelastung

	Wochenende und Feiertag Dienste<br><br>Hohes Arbeitspensum, z.T. keine persönliche Entscheidungsgewalt<br><br>vergleichsweise geringes Gehalt/Wertschätzung bei real hohem Arbeitsaufwand (ambulant vs Klinik) hohe Arbeitsbelastung mit Druck durch Leiter der Häuser<br><br>Unbefriedigend Vereinbarkeit der ärztlichen Tätigkeit mit DRG bzw. ökonomischer Druck<br><br>Zeit<br><br>Arbeitsbedingungen<br><br>Weniger Flexibilität, ggf. mangelnde Verfügbarkeit<br><br>Vergütung entspricht nicht der eines niedergelassenen Kollegen (mit Labor). Vereinbarkeit von Familie und Beruf ist schwieriger<br><br>Schichtdienst, 24h Dienste, keine Planbarkeit, kurzfristige Dienstübernahmen, schlechte Bezahlung<br><br>Arbeitsbedingung<br><br>Schichtdienst, schlechte Bezahlung,<br><br>Arbeitsbelastung, Wochenenden frei<br><br>Nachtdienste<br><br>Hohe Dienst- und Arbeitsbelastung und Wochenendarbeit<br><br>Arbeitszeiten, Dienste<br><br>nicht indizierte aufnahmen<br><br>Dienste<br><br>Nachtdienste, Wochenenddienste, nicht Chef zu sein, unflexiblere Zeitplanung<br><br>Arbeitsbelastung<br><br>Wenig Flexibilität des Arbeitsplatzes, kaum klinische Stellen verfügbar außerhalb von Uniklinika, ohne Forschung somit kaum Wahlfreiheit und auch damit nur eingeschränkt

	Dienstsystem: Feiertag-, Nachtdienst- und Wochenendtätigkeit, Schichtsystem. Fehlende Absprachemöglichkeit mit KollegInnen<br><br>Dienstsystem, in unserem Haus die Leitungsebene, mangelnde Perpektiven<br><br>Gehalt<br><br>Dienste, Fremdbestimmung durch Controlling und Personalabteilung, Personalmangel<br><br>Arbeitszeiten, Dienste, Wochenend/Feiertagsarbeit<br><br>Geringere Vergütung<br><br>Schlechte Bezahlung<br><br>Hintergrunddienste, Anstellung, zusätzliche nicht-klinische Tätigkeiten<br><br>Keine Fremdbestimmung<br><br>Dienstbelastung reduzierte Bettenzahl durch Einsparungen und zu schlechtes Widerspiegeln der Komplexitität der Fälle im DRG-System<br><br>Dienstbelastung<br><br>Unvorhersehbare Notfälle, Dienste, hohe Arbeitsbelastung, schlecht organisierte Abläufe und Kostendruck<br><br>Work-Life-Balance, Vergütung zu gering für geleistete Arbeit<br><br>Dienste und Überstunden<br><br>Dienste<br><br>Überstunden, Schichtarbeit<br><br>Schlechtere finanzielle Vergütung, schlechtere finanzielle Vergütung, schlechtere finanzielle Vergütung, schlechtere Vereinbarkeit von Familie und Beruf<br><br>Dienste, Ärger mit der Verwaltung, zu geringes Gehalt , zu lange Arbeitszeiten<br><br>Die Tatsache, dass ich auch trotz meiner Tätigkeit in der ASV Ambulanz immer werde Dienste machen müssen, um die Kollegen des Rests der Abteilung zu unterstützen.

	Dienste<br><br>Zeitliche Belastung, strukturelle Probleme, abwertung des Fachs an Kliniken, mangelnde Perspektive<br><br>Ungeregelte Arbeitszeiten, schlechtere Bezahlung<br><br>Überstunden<br><br>Bereitschaftsdienste, patriarchalische Strukturen, schlechte Bezahlung, Arbeitsverdichtung durch Personalmangel bzw. Überbelegung<br><br>Schichtarbeit, Nacht- und Wochenendedienst, schlechte work-life balance<br><br>Arbeitszeiten<br><br>Arbeits- und Dienstbelastung<br><br>Arbeitszeit<br><br>Arbeitszeit m.e. mit Familie nicht möglich ohne den Kollegen zur Last zu fallen.<br><br>Unflexible Arbeitsstrukturen, strukturelle Probleme, viel nichtärztliche Tätigkeit<br><br>Schichtarbeit, Wochenenddienste<br><br>Weniger Therapiefreiheit, Einkommen niedriger<br><br>Schichtdienst, Teilzeitmodelle oft schwierig, Überstunden/geregelte Arbeitszeit oft nicht einzuhalten.
**Bitte geben Sie die für Sie wichtigsten Argumente *für* eine Tätigkeit in der Niederlassung (als RheumatologIn) an?**	Keine Dienste<br><br>Vereinbarkeir von Familie und Beruf<br><br>Selbstständigkeit<br><br>Arbeitsumfang besser steuerbar, mehr Verdienst<br><br>Gehalt Individuellere Gestaltungsmöglichkeiten Patientenbindung Arbeitszeiten<br><br>selbstbestimmtes arbeiten

	Arbeitszeiten, Selbstbestimmung<br><br>Bessere Work-Life Balance, weniger Arbeitsbelastung<br><br>Verdienst, Eigenständigkeit, Familie&Beruf vereinbaren<br><br>Patientenbindung, Gestaltungsmöglichkeiten, eigene Ausrichtung, weniger Abhängigkeit,<br><br>Freie Wochenenden Selbstbestimmtes Arbeiten<br><br>eigener Chef sein, selbstbestimmtes Arbeiten. hoffentlich höherer Verdienst<br><br>Flexiblere Arbeitseinteilung, bessere Planbarkeit<br><br>Feste Arbeitszeite. Weniger Stress<br><br>selbstbestimmtes Arbeiten, flexiblere Arbeitsgestaltung,, geregelte Arbeitszeiten<br><br>einkommen, selbstbestimmte Arbeitszeiten<br><br>Selbständige Arbeitseinstellung, geregeltere Arbeitszeit<br><br>Vereinbarkeit mit Familie<br><br>Familienfreundlich<br><br>Selbstbestimmtes Arbeiten, Planbarkeit, großer ambulanter Bedarf an rheumatologischer Versorgung<br><br>Flexiblere Arbeitszeiteinteilung Eigenständigkeit Keine Dienste Work-Life-Balance Vergütung<br><br>Selbständigkeit Steuerung der Arbeitsbelastung<br><br>Freie Arbeitseinteilung, lange Bindung der Patienten. Selber „Chef“ sein.<br><br>Verdienst, Entscheidungsfreiheit<br><br>Bessere Arbeitszeiten, bessere Planbarkeit.<br><br>Eigenständige Zeitplanung, bessere Planbarkeit, keine Dienste<br><br>Zeit

	Keine Dienste, Arbeitszeiten, ggf. kein Vorgesetzter<br><br>arbeitszeitflexibilität, teilzeit, gehalt<br><br>Flexibilität, kontinuierliche Betreuung eigener Patienten<br><br>keine Dienste<br><br>Keine Hierarchie, Verdienstmöglichkeiten<br><br>Freizeit<br><br>Gute Organisationstruktur, Homeoffice, Vielfalt der Krankheitsbilder, langfristiger Kontakt zum Patienten<br><br>Eigenverantwortung; Selbständigkeit<br><br>Eigenverantwortlichkeit, Überstunden in Maßen, keine Nachtschichten, Flexibilät der Arbeitsgestaltung<br><br>Selbstständigkeit, freie Zeitplanung<br><br>Teamarbeit<br><br>geregelte Arbeitszeiten<br><br>Selbstbestimmtheit<br><br>keine Dienstbelastung<br><br>Freiheit bzgl Arbeitszeit, Begrenzer es Spektrum und ergo planbarere Arbeits<br><br>Autarkie<br><br>Selbstbestimmung arbeitet man viel, macht sich dies auch monetär bemerkbar<br><br>Unabhängigkeit, selbständige Entscheidungen treffen<br><br>Arbeitsbedingungen, Verdienst<br><br>Flexibilität, Gehalt<br><br>Bessere Vergütung, bessere Vereinbarkeit von Familie und Beruf<br><br>selbst festgelegte Arbeitszeiten, Geld verdienen je nach geleisteter Arbeit, der eigene Chef sein

	Bessere Arbeitsbedingungen<br><br>Eigenständigkeit, kein Schichtdienst<br><br>Wochenenden frei,<br><br>Keine Nachtdienste<br><br>flexiblere Arbeitszeiten, bessere Vereinbarkeit von Beruf und Familie - Bindung an Patienten mit besserem Vertrauensverhältnis bei regelmäßigen Konsultationen<br><br>Arbeitszeiten, weniger Dienstbelastung, planbarer<br><br>langfristige Betreuung<br><br>Fester Parientenstamm, kleiner Mitarbeiter/Kollegen Pool, Keine Dienst<br><br>Selbstständigkeit, alleine entscheiden, besseres Einkommen<br><br>meist geregelte Arbeitszeiten<br><br>Selbstständigkeit, Flexibilität, Gestaltungsfreiheit<br><br>Freie Planung der Arbeitszeit Patientenbindung und Aufbauen einer langjährigen Arzt-Patientenbeziehung<br><br>flexiblere Arbeitszeiten, keine Dienste, hoffentlich besseres Team<br><br>Selbst ständig<br><br>der eigene Chef sein, selber gestalten können, selbst das Maß der Arbeit bestimmen, Kollegen/ Personal selbst auswählen<br><br>Regelmäßige Arbeitszeiten, stressfreieres Arbeiten<br><br>Gestaltungsmöglichkeiten, guter Verdiesnt<br><br>Eigenständigkeit<br><br>Selbstständige Strukturierung, keine Hintergrunddienste<br><br>Keine Einflussnahme einer Geschäftsführung<br><br>Enge Arbeit mit Patienten regelmäßige Arbeitszeit Zielgerichtetes Arbeiten

	keine Dienstbelastung<br><br>Planbare Arbeitszeiten, höhere Verdienst, Wertschätzung<br><br>Work-Life-Balance,<br><br>flexible Arbeitszeitgestaltung, finanzielle Gründe<br><br>Vereinbarkeit Beruf und fanilie<br><br>Work-life balance<br><br>Bessere finanzielle Vergütung, bessere finanzielle Vergütung<br><br>Flexibilität, bessere Arbeitszeiten, besseres Einkommen<br><br>Die Möglichkeit flexibler Arbeitszeitgestaltung, dass man wahrscheinlich überwiegend alle Wochenenden frei haben wird<br><br>Keine Nachtdienste, mehr freie Zeit. Selbstständiges Arbeiten<br><br>Flexible Arbeitszeiten<br><br>Freiheit<br><br>Eigenständige Entscheidungen<br><br>Geregelte Arbeitszeiten<br><br>Flexiblere Arbeitszeiten, keine Dienste, bessere Verdienstmöglichkeiten.<br><br>Selbständiges Strukturieren von klinischer Arbeit und Arbeitsplatzgestaltung<br><br>Bessere work life Balance, mehr Zeit für eigene Hobbies und Projekte<br><br>Arbeitszeiten Teilzeit mgl<br><br>Flexible Arbeitsmodelle<br><br>Arbeitszeit<br><br>Planbare Arbeitszeit und planbarer Arbeitsablauf<br><br>Selbstbestimmung<br><br>Flexiblere Arbeitszeiten

	Möglichkeit zu Teilzeitmodellen, Arbeitszeitreduktion, keine Arbeit an Wochenenden oder Feiertagen.
**Bitte geben Sie die für Sie wichtigsten *gegen* eine Tätigkeit in der Niederlassung (als RheumatologIn) an?**	Zu viel Routine<br><br>Monotones arbeiten<br><br>Viel Bürokratie, praxisorganisation selten seltenes<br><br>Hoher Durchlauf an Patienten, viel Routine, wenig Zeit für Patienten<br><br>Finanzielles Risiko der Selbsständigkeit Forschen schwieriger bis kaum möglich<br><br>eigenverantwortung<br><br>Gehalt<br><br>Eher alleinstehende Arbeit<br><br>Wirtschaftliches Risiko<br><br>Gewisses unternehmerisches Risiko, weniger Austausch, höhere Patientenzahl, potentielle weniger komplexe Patient:innen<br><br>Bürokratie/Abrechnung Vertretung bei Krankheit/Urlaub<br><br>Risiko, Hoher finzanzielle Eigenbeteiligung beim Start<br><br>Man kann sich nicht so gut wie in einer Klinik weiterentwickeln.<br><br>kein direkter Austausch mit anderen Fachdisziplinen, weniger 'komplexe Fälle'<br><br>hohe Verantwortung (Personal), hohe Taktzahl, wenig Zeit für Patienten<br><br>Bürokratieaufwand, weniger interdisziplinäre Fallbesprechungen<br><br>verwaltungsaufwand<br><br>Fehlende diagnostische Möglichkeiten<br><br>Kleines Team<br><br>Zulassungsbeschränkung, mangelnder Austausch mit Kolleg:innen ('kochen der eigenen Suppe')

	Viele Arthrosen, wenig Arthritis Schlechtere interdisziplinäre Zusammenarbeit Mehr Koordinationsaufwand<br><br>Einschränkung der Diagnostik<br><br>Komplizierterer kollegialer Austausch.<br><br>Oft Einzelkämpfer, Budgetplanung, eigene Abrechnung<br><br>Risiko, Arbeitszeiten<br><br>Der hohe Arbeitsvolumen und zeitlicher Aufwand.<br><br>Komplizierter Wechsel von Klinik in Niederlassung<br><br>Team<br><br>Finanzielles Risiko, weniger interdisziplinäres Arbeiten, weniger fachlicher Austausch, weniger komplexe Fälle<br><br>arbeitsdichte, weniger zeit pro patient, weniger kontakt zur nephro, pulmo etc.<br><br>Höheres wirtschaftliches Risiko<br><br>Money rules everything - Abfertigung von Patienten<br><br>Kaum verfügbare KV-Sitze („überversorgt“)<br><br>keine Forschung<br><br>hohes finanzielles Risiko, ökonomische Zwänge, Angst vor Regressvorderungen<br><br>gibt keine<br><br>Therapie und Diangosemöglichkeiten beschränkt, kein Angestelltenverhältnis (Mutterschutz/Krankheit)<br><br>privates Risiko im Falle von Krankheit<br><br>Routine, auf sich alleine gestellt sein<br><br>weniger interessante Fälle<br><br>Verwaltung, Abrechnung

	Verantwortung Krankheitsbilder<br><br>finanzielle Risiken, Zwänge und Limitationen<br><br>hoher bürokratischer Aufwand Ärger mit der KV DD Regress<br><br>Unsichere Situation hinsichtlich ökonomischer Entwicklungen<br><br>Interdisziplinärer Austausch fehlt<br><br>mehr Patientenfälle, dafür ggf. weniger komplex, wirtschaftliches Arbeiten<br><br>häufigere Routinefälle. Komplexe Probleme müssen an die stationäre Versorgung abgegeben werden.<br><br>fehlende Ausbildung des Nachwuchses<br><br>Wg Ärztemangel nur noch Zeit für das allernötigste über den Tellerrand denken zeitlich kaum mehr möglich, da die Fallzahlen stimmen müssen<br><br>Allein arbeiten<br><br>Weniger spezielle Fälle, geringere Möglichkeit an diagnostik<br><br>Weniger Zeit für die Patienten<br><br>Weniger intensive Auseinandersetzung, schwere Fälle werden stationär verwiesen, viel Routine<br><br>komplex<br><br>Wenig spannend<br><br>keine Grundlagenforschung möglich<br><br>Patientenkollektiv, Finanzielles Risiko<br><br>Ggf. Monotonie<br><br>Sicherheit des Arbeitesplatzes Ökonomisches Denken Fehlende Teamarbeit, fehlende Fallbesprechungen<br><br>die Weiterbildungszeit (für die Rheumatologie) in der Klinik, hohe Belastung durch Regressdrohung und zu viele Patient*innenanfragen<br><br>Politik

	'Einzelkämpferdasein', viele zusätzliche Verpflichtungen (Buchhaltung, IT, etc.), schwerer Informationszugang bei der KV, viel Unklarheit was einen erwartet und wie man es wirtschaftlich gestaltet, mangelnde betriebswirtschaftliche Ausbildung<br><br>Wenig Abwechslung, viel Routinearbeit<br><br>Papierkram/ administrative Aufgaben in hohem Maße<br><br>Finanzieller Druck<br><br>Wirtschaftliches Risiko<br><br>Ambulantes Arbeiten<br><br>Risiko der Selbstständigkeit Monotonie<br><br>geringere Möglichkeiten an Diagnostik und Therapie sowie weniger interessante Fälle<br><br>Fachliche Vereinsamung, wenig Weiterbildung<br><br>weniger Diagnostikmöglichkeiten, weniger Austausch mit anderen Fachrichtungen<br><br>Monotonie, Gebührenordnung<br><br>Vielleicht evtl monotones Arbeit<br><br>Langweiligere Arbeit<br><br>Risiko der Eigenverantwortung<br><br>Ein möglicher Verdienstverlust, dass man sich in der KV Versorgung leider für die komplexen und teilweise psychisch sehr belasteten Rheuma Patienten leider oft nicht ausreichend Zeit nehmen kann.<br><br>Einseitigkeit, hoher Durchsatz mit wenig Zeit für Patienten:innen<br><br>Bürokratie<br><br>Routine<br><br>Fachlicher Austausch fehlt, fehlende KV Sitze<br><br>Fehlen von Interdisziplinarität, größerer wirtschaftlicher Druck

	Selbständigkeit, multidisziplinäre Arbeit sehr eingeschränkt<br><br>Langweilig<br><br>Weniger spannende Fälle, viel Routine<br><br>Rheumatologie als alleiniges Fach für mich nicht interessant.<br><br>Personalverantwortung, wenig Zeit für den Patientenkontakt<br><br>kleines Team, weniger Kollegen, ggf.weniger Besprechungsmöglichkeiten<br><br>Teils hohe Arbeitsverdichtung.

Tabelle 13: Berufsleben als Facharzt/Fachärztin für Innere Medizin und Rheumatologie.

**Work-Life-Balance**	1 (sehr wichtig)	n = 38 (37,3 %)
	2	n = 33 (32,4 %)
	3	n = 9 (8,8 %)
	4	n = 8 (7,8 %)
	5	n = 5 (4,9 %)
	6	n = 5 (4,9 %)
	7	n = 4 (3,9 %)
	8 (unwichtig)	n = 0 (0 %)
**Teilzeittätigkeit**	1 (sehr wichtig)	n = 16 (15,7 %)
	2	n = 12 (11,8 %)
	3	n = 11 (10,8 %)
	4	n = 12 (11,8 %)
	5	n = 13 (12,7 %)
	6	n = 12 (11,8 %)
	7	n = 9 (8,8 %)
	8 (unwichtig)	n = 17 (16,7 %)
**Vereinbarkeit von Familie und Beruf**	1 (sehr wichtig)	n = 44 (43,1 %)
	2	n = 28 (27,5 %)
	3	n = 9 (8,8 %)
	4	n = 8 (7,8 %)
	5	n = 5 (4,9 %)
	6	n = 3 (2,9 %)
	7	n = 5 (4,9 %)
	8 (unwichtig)	n = 0 (0 %)
**Klinische Tätigkeit**	1 (sehr wichtig)	n = 26 (25,5 %)
	2	n = 20 (19,6 %)
	3	n = 22 (21,6 %)
	4	n = 12 (11,8 %)
	5	n = 5 (4,9 %)
	6	n = 8 (7,8 %)
	7	n = 8 (7,8 %)
	8 (unwichtig)	n = 1 (1,0 %)
**Selbstständigkeit**	1 (sehr wichtig)	n = 14 (13,7 %)
	2	n = 20 (19,6 %)
	3	n = 18 (17,6 %)
	4	n = 13 (12,7 %)
	5	n = 10 (9,8 %)
	6	n = 11 (10,8 %)
	7	n = 7 (6,9 %)

	8 (unwichtig)	n = 9 (8,8 %)
**Regionale Bindung**	1 (sehr wichtig)	n = 14 (13,7 %)
	2	n = 13 (12,7 %)
	3	n = 16 (15,7 %)
	4	n = 17 (16,7 %)
	5	n = 12 (11,8 %)
	6	n = 11 (10,8 %)
	7	n = 10 (9,8 %)
	8 (unwichtig)	n = 9 (8,8 %)
**Keinen Wochenend-/ Nachtdienst**	1 (sehr wichtig)	n = 39 (38,2 %)
	2	n = 22 (21,6 %)
	3	n = 11 (10,8 %)
	4	n = 9 (8,8 %)
	5	n = 5 (4,9 %)
	6	n = 6 (5,9 %)
	7	n = 1 (1,0 %)
	8 (unwichtig)	n = 9 (8,8 %)
**Verdienstmöglichkeiten**	1 (sehr wichtig)	n = 23 (22,5 %)
	2	n = 26 (25,5 %)
	3	n = 16 (15,7 %)
	4	n = 16 (15,7 %)
	5	n = 12 (11,8 %)
	6	n = 1 (1,0 %)
	7	n = 4 (3,9 %)
	8 (unwichtig)	n = 4 (3,9 %)

Tabelle 14: Mitgliedschaften in Fachgesellschaften.

**Sind Sie ein Mitglied der Deutschen Gesellschaft für Rheumatologie?**	Nein	n = 30 (29,4 %)
	Ja	n = 70 (70,6 %)
**Sind Sie Mitglied im Berufsverband Deutscher Rheumatologen?**	Nein	n = 96 (94,1 %)
	Ja	n = 6 (5,9 %)
**Sind Sie Mitglied in der AGJR?**	Nein	n = 56 (54,9 %)
	Ja	n = 46 (45,1 %)

7 Praktische Tätigkeiten

Tabelle 15: Mentoring und Diagnostik im Rahmen der Facharztausbildung.

**Werden praktische Tätigkeiten am Arbeitsplatz durch einen Mentor (Facharzt) betreut?**	Nein<br>Ja	n = 41 (40,2 %)<br>n = 61 (59,8 %)
**Führen Sie Arthrosonographien *unter Anleitung* durch?**	Nein<br>Ja	n = 60 (58,8 %)<br>n = 42 (41,2 %)
**Führen Sie *selbstständig* Arthrosonographien durch?**	Nein<br>Ja	n = 14 (13,7 %)<br>n = 88 (86,3 %)
**Bei wie vielen Patienten pro Monat führen Sie *selbstständig* Arthrosonographien durch?**	<5<br>5-10<br>11-20<br>20-50<br>>50	n = 14 (15,9 %)<br>n = 24 (27,3 %)<br>n = 22 (25,0 %)<br>n = 18 (20,5 %)<br>n = 10 (11,1 %)
**Führen Sie Gefäßsonographien *unter* Anleitung durch?**	Nein<br>Ja	n = 66 (64,7 %)<br>n = 36 (35,3 %)
**Führen Sie *selbständig* Gefäßsonographien durch?**	Nein<br>Ja	n = 58 (56,9 %)<br>n = 44 (43,1 %)
**Wie viele Gefäßsonographien führen Sie *selbstständig* pro Monat durch (Angabe einer Zahl)?**	<5<br>5-10<br>11-20<br>20-50<br>>50	n = 22 (50,0 %)<br>n = 16 (36,4 %)<br>n = 5 (11,4 %)<br>n = 1 (2,3 %)<br>n = 0 (0 %)
**Führen Sie Gelenkpunktionen *unter* Anleitung durch?**	Nein<br>Ja	n = 40 (39,2 %)<br>n = 62 (60,8 %)
**Führen Sie *selbständig* Gelenkpunktionen durch?**	Nein<br>Ja	n = 27 (26,5 %)<br>n = 75 (73,5 %)
**Wie viele Gelenkpunktionen führen Sie selbstständig pro Monat durch (Angabe einer Zahl)?**	<5<br>5-10<br>11-20<br>20-50<br>>50	n = 37 (49,3 %)<br>n = 20 (26,7 %)<br>n = 14 (18,7 %)<br>n = 4 (5,3 %)<br>n = 0 (0 %)

**Führen Sie Kapillarmikroskopien *unter* Anleitung durch?**	Nein<br>Ja	n = 72 (70,6 %)<br>n = 30 (29,4 %)
**Führen Sie *selbständig* Kapillarmikroskopien durch?**	Nein<br>Ja	n = 46 (45,1 %)<br>n = 56 (54,9 %)
**Wie viele Kapillarmikroskopien führen Sie selbstständig pro Monat durch (Angabe einer Zahl)?**	<5<br>5-10<br>11-20<br>20-50<br>>50	n = 27 (48,2 %)<br>n = 23 (41,1 %)<br>n = 4 (7,1 %)<br>n = 0 (0 %)<br>n = 2 (3,6 %)

Tabelle 16: Labor und immunologische Diagnostik im Rahmen der Facharztausbildung.

**Besteht an Ihrer Weiterbildungsstätte (oder einer assoziierten Einrichtung) die Möglichkeit zum Erwerb der „Handlungskompetenz“ (=Anordnung, Kenntnisse zur Durchführung, Validierung der Befunde und Supervision der Durchführung) für die rheumatologische/immunologische Labordiagnostik?**	Nein<br>Ja	n = 47 (46,1 %)<br>n = 55 (53,9 %)
**Besitzen Sie nach eigener Einschätzung die „Handlungskompetenz“ (=Anordnung, Kenntnisse zur Durchführung, Validierung der Befunde und Supervision der Durchführung) für die rheumatologische/immunologische Labordiagnostik?**	Nein<br>Ja	n = 71 (69,6 %)<br>n = 31 (30,4 %)
**Wie schätzen Sie Ihren Wissensstand die Qualität Ihrer praktischen Fertigkeiten in der rheumatologisch/immunologischen Labordiagnostik ein?**	nicht vorhanden<br>geringe Kenntnisse / Fertigkeiten<br>Grundkenntnisse<br>Fortgeschrittene Kenntnisse / Fertigkeiten	n = 8 (7,8 %)<br>n = 36 (35,3 %)<br>n = 43 (42,2 %)<br>n = 15 (14,7 %)

Tabelle 17: Fähigkeiten und Kenntnisse in der Osteodensitometrie.

**Stellen Sie die Indikation zur Durchführung von Osteodensitometrie und befunden diese eigenständig?**	Nein<br>Ja	n = 38 (37,3 %)<br>n = 64 (62,7 %)
**Wie viele Osteodensitometrien werten Sie pro Monat aus?**	<5<br>5-10<br>11-20<br>20-50<br>>50	n = 51 (50,0 %)<br>n = 21 (20,6 %)<br>n = 18 (17,6 %)<br>n = 9 (8,8 %)<br>n = 3 (2,9 %)

Tabelle 18: Weiterbildung im Bereich der bildgebenden Verfahren.

**Werden regelmäßige interdisziplinäre radiologische Fallbesprechung durchgeführt?**	Nein<br>Ja	n = 26 (25,5 %)<br>n = 76 (74,5 %)
**Erhalten Sie eine Ausbildung in der Interpretation von Röntgenbildern?**	Nein<br>Ja	n = 53 (52,0 %)<br>n = 49 (48,0 %)
**Erhalten Sie eine Ausbildung in der Interpretation von CT-Bildern?**	Nein<br>Ja	n = 66 (64,7 %)<br>n = 36 (35,3 %)
**Erhalten Sie eine Ausbildung in der Interpretation von MRT-Bildern?**	Nein<br>Ja	n = 63 (61,8 %)<br>n = 39 (38,2 %)
**Erhalten Sie eine Ausbildung in der Interpretation von nuklearmedizinischen bildgebenden Verfahren (z. B. Skelettszintigraphie, Speicheldrüsenszintigraphie, PET/CT, PET/MRT)?**	Nein<br>Ja	n = 72 (70,6 %)<br>n = 30 (29,4 %)

8 Persönliche Meinung / Kommentare

Tabelle 19: Persönliche Meinung / Kommentare.

**Was ist der Grund (Gründe), dass Sie eine Facharztausbildung im Fachgebiet Innere Medizin und Rheumatologie aufgenommen haben?**	Interessantes fach<br><br>Spannendes Patientenkollektiv<br><br>Das breite Spektrum an Erkrankungen, Immunologie und Forschung an immunologischen Hintergründen der Erkrankungen<br><br>Inhaltliches Interesse am Fach, Vielfalt der Inhalte<br><br>Natürlich vor allem, dass es ein interessantes Fachgebiet und dass es mir persönlich einigermaßen leicht von der Hand geht, die gute Möglichkeit der Niederlassung<br><br>work/life balance, fachliches interesse<br><br>Vorlesung in der Uni<br><br>Super spannendes Fach, tolle Forschung, flache Hierarchien, familiäres Setting unter Kolleg:innen<br><br>Spannendes Fach, praktische Fertigkeiten(Sono!)<br><br>Sehr komplexe Krankheitsbilder mit der verhältnismäßig hohen Wahrscheinlichkeit helfen zu können. Frühes ambulantes Arbeiten Weniger Nacht-und Wochenenddienste Geregelteres Leben Leidenschaft für die Medizin<br><br>Breites internistisches Fach mit hohem Anteil interdisziplinären Arbeitens Diagnostische und therapeutische Möglichkeiten Affinität zu Sonographie<br><br>Interesse an klinischer Immunologie<br><br>Interesse an der Immunologie<br><br>Es ist ein spanendes Gebiet mit viele neue Therapiemöglichkeiten.<br><br>spannendes Berufsbild, persönliches Interesse<br><br>Interesse am Fach.+

	Große Vielfalt an Krankheitsbildern, interdisziplinäre Behandlung der Patienten,<br><br>hat sich so ergeben<br><br>Fachliches Interesse<br><br>Spannende Fälle<br><br>Nettes, Team, spannende Medizin, kognitiv anspruchsvoll<br><br>Spannendes Fachgebiet<br><br>Komplexität und Vielfalt rheumatologischer Krankheitsbilder<br><br>Interessante, differentialdiagnostische Fälle<br><br>Weites Fachgebiet mit einem abwechslungsreichen Spektrum. Patienten in allen Lebensdekaden. „Etwas“ ruhiger als andere Gebiete ;-) man hat mehr Zeit zum Nachdenken und diskutieren. Ein Fach für Sonoliebhaber. :-)<br><br>Interessantes Fach<br><br>Spannendes medizinisches Gebiet mit viel therapeutisches Potenzial aktuell und für die Zukunft.<br><br>Komplexe Krankheitsbilder, wissenschaftlicher Fortschritt, klinisches Arbeiten, interdisziplinäre Besprechungn<br><br>Vielfalt<br><br>Interessantes Fach, breit, divers, unterschiedlichste Patientenkollektive, gute Begandlungserfolge, dynamisches Fach mit spannenden therapeutischen Entwicklungen<br><br>Interdisziplinäre, buntes klientel<br><br>Spannendes Fach, Kollegialität, Sehr gute Betreuung während PJ<br><br>Das Fach 'brennt' - es ist unglaublich interessant, es finden viele Innovationen statt, es ist schlau und mitten in der Inneren Medizin<br><br>Interesse am Fachgebiet

	umfassendes Wissen erforderlich, um komplexe Zusammenhänge zu erfassen; jedes Organsystem ist einbezogen<br><br>Krankheitsbilder, Immunologische Hintergründe, immer wieder sehr spannende Fälle<br><br>Forschung, Grunderkrankungen, Therapeutische Möglichkeiten<br><br>viele neue Forschungen und Therapiemöglichkeiten - relativ junge Patienten - Multidisziplinarität der Medizin<br><br>Begeisterung für das Fach<br><br>Interesse am Fach<br><br>Das für mich interessanteste Fach<br><br>Persönliches Umfeld, spannendes Fachgebiet<br><br>PJ<br><br>politische Gründe<br><br>Die Liebe und Leidenschaft zum Fachgebiet<br><br>Seltene Erkrankungen<br><br>Super Fachrichtung<br><br>Interesse für das Fach und die fachbezogene Forschung<br><br>Ich liebe dieses vielseitige Fach mit seiner unglaublich interessanten Differenzialdiagnostik und den großen Überschneidungen mit diversen anderen medizinischen Disziplinen. Das Patientenklientel ist zudem vielschichtig und leidet meist schicksalhaft an einer Autoimmunerkrankung<br><br>Gute Vorlesung in der Uni<br><br>Tolles abwechslungsreiches Fach :)<br><br>Interdiziplinarität, Rheumatologen werden gebraucht

	Nahe am Patienten, viel Medizin mit wenig maschinellen Voraussetzungen möglich.<br><br>klm<br><br>Querschnittsbereich mit vielfältiger Organbeteiligung, interessant, Weiterentwicklung von Diagnostik und Therapie auch in Zukunft<br><br>Spannend<br><br>Spannend<br><br>Die Elternzeitfrage kann man nicht beantworten. Ich hatte 2 Monate, nicht 100<br><br>extensive Diagnostik mit meist guten Behandlungsmöglichkeiten<br><br>Spannendes Arbeitsgebiet. Hilfe leisten wo viel bedarf besteht<br><br>Interesse und Vielseitigkeit der Erkrankungen, Möglichkeit der langjährigen Betreuung von PatientInnen<br><br>Ich finde das Immunsystem sehr spannend und hätte mir gewünscht, in dem Feld zu arbeiten, da ich zu Autoimmunität in meiner Doktorarbeit geforscht habe und das sehr spannend fand. Ich wollte Forschung und Lehre machen und dachte, ich könnte das in unserer Fachabteilung ggf. in der Kombination Rheumatologie/Infektiologie machen.<br><br>Breite Innere Medizin<br><br>Spaß am Detektiv sein, viel Entwicklungspotential<br><br>Interessantes Fachgebiet<br><br>Spannende Kasuistiken, keine Nachtdienste/Notfälle als Hintergrund im Krankenhaus, hohes Maß der Interdisziplinarität, Menschen als Ganzes sehen<br><br>Fachlicher Inhalt<br><br>- Interdisziplinarität - Differentialdiagnostik - Komplexe Fälle - Patient:in und Lebensqualität im Fokus<br><br>Spannendes, weitgreifendes Gebiet, breite Patientenklientel

	Liebe zum Fach,<br><br>Interessantes Fach mit sehr breiten Überschneidungen<br><br>Schnittstelle zur Nephrologie<br><br>Aus meiner Sicht fachlich von allen internistischen Disziplinen am interessantesten<br><br>Persönliches Interesse an rheumatologischen Erkrankungen, nettes Arbeitsumfeld in meiner Klinik, Mangel an Rheumatologinnen<br><br>Gutes Team, interessante Fälle<br><br>Interessante Krankheitsbilder, viele wirksamen Therapieoptionen, wenig Notfälle<br><br>Rheumatologie ist ein äußerst vielseitiges und spannendes Fach mit meist angenehmen Patienten jedes Alters, die meistens schicksalhaft, ohne Verschulden erkranken. Mich reizt die vielseitige Differenzialdiagnostik, das Detektivspiel auf dem Weg zur richtigen Diagnose - insbesondere bei komplexen Erkrankungen<br><br>Fachliches Interesse, nettes Team<br><br>Den Facharzt für Innere Medizin Habenichtse gemacht, weil ich es das spannendste und vielfältigste Fach mit viel Detektivarbeit finde - Die Rheumatologie interessiert mich wegen der Komplexität der Krankheitsbilder und der ständigen Weiterentwicklung des Verständnisses der Erkrankungen und Therapiemöglichkeiten<br><br>intereses am Fach<br><br>Interesse, Vielseitigkeit des Fachs, Therapieziel des Leidensminderns und bessern der Lebensqualität der Behandelten<br><br>Tolles Team, spannendes Fachgebiet<br><br>Interesse, Komplexität<br><br>Interesse für das Fachgebiet<br><br>Interdisziplinäres Arbeiten, Komplexe internistische Fälle<br><br>Interesse für das Fach, Rheuma ist zu großen Teilen ein ambulantes Fach

	PJ spannende fälle<br><br>Interesse am fach<br><br>Spannendes fach<br><br>Vorheriges Studium der Biologie mit Schwerpunkt Immunologie. Daher Grundwissen über Pathomechanismen und Vertsändnisn und Interesse für den Wirkmechan. der Therapien.<br><br>Fachliches Interesse<br><br>Interessantes Fachgebiet, Erweiterung des Horizonts zur (allgemein)internistischen Ausbildung, in der die Rheumatologie kaum gelehrt wird<br><br>DGrh Studentenprogramm<br><br>Interesse am Fachgebiet	
**Haben sich Ihre oben genannten geäußerten Erwartungen (vorherige Frage) erfüllt?**	Nein<br><br>Ja	n = 11 (10,8 %)<br><br>n = 91 (89,2 %)
**Was schätzen Sie am Fachgebiet Innere Medizin und Rheumatologie?**	Die Kollegen und die Patienten<br><br>Das breite Spektrum der Erkrankungen jegliches Organsystem betreffend, Immunologie verstehen lernen<br><br>Spannende Fälle, in der Regel wenig Schichtarbeit<br><br>Besonders schätze ich, dass man in der Niederlassung trotzdem ein sehr weites Spektrum an Erkrankungen weiter behandelt(im Gegensatz z.b. zur Kardiologie, Unfallchirurgie, Urologie...), außerdem der gute Zusammenhalt unter Rheumatologen<br><br>Fortschritt der Forschung<br><br>Komplexe Krankheitsbilder, Seltene Erkrakungen, praktische Tätigkeiten<br><br>Community-Gedanke mit Hilfsbereitschaft zwischen den verschiedenen Standorten. Von allen internistische Gebieten das Wesentliche im Alltag zu überblicken. Ambulantes Arbeiten. Für die Innere eine nicht so desaströse Work-life-balance.	

	Breites internistisches Fach mit hohem Anteil interdisziplinären Arbeitens Diagnostische und therapeutische Möglichkeiten Affinität zu Sonographie<br><br>unteressante Fälle<br><br>abwechslungsreiche Krankheitsbilder<br><br>Die Zufriedenheit des Patienten.<br><br>komplexe Diagnostik, gute Therapiemöglichkeiten, Arbeit mit Patient:innen, interdisziplinäre Zusammenarbeit im Team<br><br>breites fach<br><br>Patienten und komorbiditäten werden behandelt, nicht nur die eine Diagnose. Breites Basiswissen der inneren Medizin erforderlich.<br><br>komplexizität patientenorientiert<br><br>Interdisziplinäres Gebiet<br><br>Unterschiedliches Patientengut, viel eigenständige Diagnostik möglich<br><br>Vielseitigkeit, keine Organmedizin, sympathische Kolleg:innen<br><br>Die klinische Arbeit sowie die Krankheitsbilder Möglichkeit einer Niederlassung<br><br>Siehe 8.1. Die breite Aufstellung und das interdisziplinäre Arbeiten.<br><br>Ambulante Tätigkeit, fachliche Herausforderung<br><br>Kleineres Fach mit viel Potenzial und breites Altersspektrum der Patienten.<br><br>Patientenorientiertes Arbeiten, interdisziplinäre Besprechungen<br><br>Team<br><br>Siehe oben<br><br>spannend<br><br>Breites Spektrum an Erkrankungen und Organmanifestationen, abwechslungsreich

	siehe oben
	Kollegen, Fortschrittlichkeit, Patientengut, Inhalte fachlich, Vereinbarkeit mit der Familie
	Spektrum der Erkrankungen, Innovationen insbesondere der Therapie jedes Jahr; fachübergreifendes Arbeiten und Wissen
	interdisziplinäres Denken, spitzfindiges Denken in Bezug auf seltene Syndrome, fachliche Überlappung zu Pädiatrie/Neurologie/Nephrologie/Hämatologie, Möglichkeiten der immunologischen Diagnostik
	Interaktion mit Patienten, Weiterentwicklung von Diagnostik und Therapie
	Die Grundkentniss in der inneren Medizin ist hier sehr wichtig.
	Vielfalt der Krankheitsbilder
	Komplexität der Fälle, langfristiger Patientenkontakt, schnelle Weiterentwicklung
	Die Breite des Faches
	Interdisziplinäres Arbeiten
	Spannende Krankheitsbilder, ganzheitlich Untersuchung der Patienten
	easy customer satisfaction and retention
	Interdisziplinarität
	Die vielfältigen Erkrankungen und die Innere Medizin als solches, da Rheumatologie internistisch fachübergreifend ist.
	Komplexität
	Interdisziplinarität, spannende Krankheitsbilder
	Ich liebe dieses vielseitige Fach mit seiner unglaublich interessanten Differenzialdiagnostik und den großen Überschneidungen mit diversen anderen medizinischen Disziplinen. Das Patientenklientel ist zudem vielschichtig und leidet meist schicksalhaft an einer Autoimmunerkrankung

	Gute Jobaussichten aufgrund des Mangels, spannendes Fach, viel Forschungspotential<br><br>Detektivarbeit & die Möglichkeit oft helfen zu können<br><br>Interdisziplinarität, 'Detektivarbeit'<br><br>Spannend, viel nachdenken<br><br>Spannend, Überschneidungen mit vielen anderen Fachgebieten, rasante Entwicklung der Therapiemöglichkeiten, Stellenwert der Anamnese und der guten Arzt-Patienten-Beziehung.<br><br>Querschnittsbereich mit vielfältiger Organbeteiligung, interessant, Weiterentwicklung von Diagnostik und Therapie auch in Zukunft<br><br>Ganzheitliche Betreuung von Patientinnen<br><br>Spannend<br><br>Spannend<br><br>Zusammenhalt, Interdisziplinarität, chronische Erkrankungen mit Reevaluationen im Verlauf<br><br>Besonderes Fachgebiet, kleine Facharzt Gemeinde, Wertschätzung anderer Fächer.<br><br>Vielfältigkeit, Interdisziplinärer Ansatz, Ganzheitliche Betrachtung des internistischen Patienten, breite Altersspanne, kollegiale Zusammenarbeit, weites Forschungsfeld<br><br>Ich finde weiterhin das Immunsystem und seine Komplexität sehr spannend. Es gibt einen ganzheitlichen Ansatz, den ich sehr schätze. Desweiteren mag ich die Verzahnung mit Infektiologie sowie Psychosomatik/Schmerzmedizin. Die Krankheiten sind sehr vielgestaltig und dadurch anspruchsvoll und spannend.<br><br>Interdisziplinäre ZA<br><br>sehr dynamisches Fachgebiet mit Zukunftsperspektive, interdisziplinäre Zusammenarbeit mit vielen Fachrichtungen, sehr freundliche und unkomplizierte Fachkollegen deutschland- und europaweit ... dass es jedes Mal anders ist und kein Pat. wie der andere

	Kompetenz und breites Wissen der Inneren Medizin und Rheumatologie<br><br>Abwechslung<br><br>Immer wieder neuer Wissenszugewinn<br><br>Abwechslungsreiche komplexe Krankheitsbilder mir stetem Blick über den Tellerrand<br><br>Vielfalt an klinischen Krankheitsbildern<br><br>Spannende Differentialdiagnosen<br><br>Das Patientenklientel, von jung-alt, krank bis weniger krank, Möglichkeiten, sowohl stationär als auch ambulant zu arbeiten.<br><br>Überschneidung zu anderen internistischen Fachgebieten, Patienten jeglichen Alters und Geschlechts, gute Therapiemöglichkeiten<br><br>Viel phienzennahe Tätigkeit<br><br>Langfristige Kontakt mit Patienten, work-life balance<br><br>Rheumatologie ist ein äußerst vielseitiges und spannendes Fach mit meist angenehmen Patienten jedes Alters, die meistens schicksalhaft, ohne Verschulden erkranken. Mich reizt die vielseitige Differenzialdiagnostik, das Detektivspiel auf dem Weg zur richtigen Diagnose - insbesondere bei komplexen Erkrankungen<br><br>die Diagnostik kann eine Herausforderung sein<br><br>Interesantes fach<br><br>Vielseitigkeit, Faszination, interdisziplinarität<br><br>Spannendes Fachgebiet<br><br>Therapiemöglichkeiten, Komplexität<br><br>Man erhält gute Ergebnisse bei erfolgreicher Therapie, Vielseitigkeit, dankbare Patienten. Die Pflicht zu 6 Monate Intensivmedizin sollte überdacht werden. Diese hält Kolleg*innen von der FA Weiterbildung ab.

	Interessant und insgesamt befriedigend. Die Hauptprobleme liegen an der (Uni-)Klinik und nicht an dem Fach<br><br>Spannende Fälle<br><br>Breites Spektrum an Patienten<br><br>Ambulant und spannend<br><br>Abwechslunsgreich, in dr rheumatologischen Ambulanz sicherlich sinnvoll für spätere niedegrelassene Tötigkeit<br><br>neue Therapieoptionen, langjährige Betreuung<br><br>Jüngere Patienten, Erfolg bei Therapien<br><br>Langfristige Patientenbetreuung<br><br>Breites Spektrum an Krankheitsbildern, mentale Herausforderung, ganzheitliches Patientenverständnis.
